# Pathologic and Phenotypic Alterations in a Mouse Expressing a Connexin47 Missense Mutation That Causes Pelizaeus-Merzbacher–Like Disease in Humans

**DOI:** 10.1371/journal.pgen.1002146

**Published:** 2011-07-07

**Authors:** Oliver Tress, Marta Maglione, Armin Zlomuzica, Dennis May, Nikolai Dicke, Joachim Degen, Ekrem Dere, Helmut Kettenmann, Dieter Hartmann, Klaus Willecke

**Affiliations:** 1 Institute of Genetics, Division of Molecular Genetics, University of Bonn, Bonn, Germany; 2 Cellular Neurosciences, Max-Delbrück Center for Molecular Medicine, Berlin, Germany; 3 Center for the Study and Treatment of Mental Health, Ruhr-Universität Bochum, Bochum, Germany; 4 Université Pierre et Marie Curie (Paris VI), UMR 7102, Neurobiologie des Processus Adaptatifs, Paris, France; 5 Department of Anatomy, Division of Neuroanatomy, University of Bonn, Bonn, Germany; The Jackson Laboratory, United States of America

## Abstract

Gap junction channels are intercellular conduits that allow diffusional exchange of ions, second messengers, and metabolites. Human oligodendrocytes express the gap junction protein connexin47 (Cx47), which is encoded by the *GJC2* gene. The autosomal recessive mutation hCx47M283T causes Pelizaeus-Merzbacher–like disease 1 (PMLD1), a progressive leukodystrophy characterized by hypomyelination, retarded motor development, nystagmus, and spasticity. We introduced the human missense mutation into the orthologous position of the mouse *Gjc2* gene and inserted the *mCx47M282T* coding sequence into the mouse genome via homologous recombination in embryonic stem cells. Three-week-old homozygous *Cx47M282T* mice displayed impaired rotarod performance but unchanged open-field behavior. 10-15-day-old homozygous *Cx47M282T* and Cx47 null mice revealed a more than 80% reduction in the number of cells participating in glial networks after biocytin injections into oligodendrocytes in sections of corpus callosum. Homozygous expression of *mCx47M282T* resulted in reduced MBP expression and astrogliosis in the cerebellum of ten-day-old mice which could also be detected in Cx47 null mice of the same age. Three-month-old homozygous *Cx47M282T* mice exhibited neither altered open-field behavior nor impaired rotarod performance anymore. Adult *mCx47M282T* expressing mice did not show substantial myelin alterations, but homozygous *Cx47M282T* mice, additionally deprived of connexin32, which is also expressed in oligodendrocytes, died within six weeks after birth and displayed severe myelin defects accompanied by astrogliosis and activated microglia. These results strongly suggest that PMLD1 is caused by the loss of Cx47 channel function that results in impaired panglial coupling in white matter tissue.

## Author Summary

Oligodendrocytes are the myelinating cells of the central nervous system. Together with astrocytes, oligodendrocytes form networks of coupled glial cells—so-called panglial networks—which are built by gap junctions, i.e. intercellular channels composed of connexin proteins. Pelizaeus-Merzbacher–like disease is an inherited early onset myelin disorder of the central nervous system. Certain mutations of the connexin47 gene, which is expressed by oligodendrocytes, cause this disease. Although the course of the human disease is conspicuous and progressive, connexin47 null mice do not show obvious phenotypic alterations suggesting that the disease may be caused by gain of detrimental function due to the connexin47 mutations. Here we introduced a missense mutation that was found in Pelizaeus-Merzbacher–like disease patients into the connexin47 mouse gene. Expression of the mutant connexin47 gene in oligodendrocytes resulted in myelin malformations in young mice but to a relatively mild extent. From a comparison of connexin47 null and connexin47 mutant mice, we conclude that the human Pelizaeus-Merzbacher–like disease is caused by loss of gap junctional coupling, which results in a decreased number of cells coupled within glial networks, and not by a gain of detrimental function of the mutated protein.

## Introduction

The autosomal, recessively inherited Pelizaeus-Merzbacher-like disease 1 (PMLD1; MIM: 608804) is an early onset hypomyelinating leukodystrophy caused by mutations in the human connexin47 (Cx47) gene *GJC2* (previously called *GJA12*; MIM: 608803) [1]. Like X-linked Pelizaeus-Merzbacher disease (PMD, MIM: 312080), which is caused by mutations in the gene encoding proteolipid protein 1 (*PLP1*, MIM: 300401), one of the major proteins in the central nervous system (CNS) myelin, PMLD is characterized by impaired motor development resulting in nystagmus, dysarthria, progressive spasticity and ataxia. First symptoms, nystagmus and poor control of head and trunk movements, occur during early infancy. Twenty-four different mutations including missense, nonsense, partial deletion and frameshift mutations of the *GJC2* gene have been reported for PMLD-affected patients to date [1]–[8]. The milder late onset hereditary spastic paraplegia (SPG44, MIM: 613206) is associated with another recessive missense mutation *I33M* in the *GJC2* gene [9].

All PMLD1 patients are homozygous or compound heterozygous for mutations of the *GJC2* gene. Neurological symptoms or MRI abnormalities were not detected in heterozygous individuals [1]–[3] but dominantly inherited lymphedemas (MIM: 613480) were recently described to be associated with *GJC2* mutations [10].

The *GJC2* gene encodes the gap junction protein Cx47. Gap junction channels (GJCs) are intercellular conduits for diffusional exchange of ions and small molecules like metabolites and second messengers. Each of the apposed cells contribute per GJC one connexon (hemichannel) which consists of six connexin proteins. Twenty-one human connexins and 20 rodent connexins were described so far which adds to the great theoretical diversity of gap junction channels, since connexons may be composed of one (homomeric) or more than one (heteromeric) connexin isoform. Coupling of connexons consisting of different connexin isoforms is referred to as heterotypic coupling, in contrast to homotypic coupling resulting from GJCs composed of the same connexin isoform. However, the diversity for GJCs is limited because not all heterotypic channels appear to be functional in cultured cells [11], and different cell types express only few connexin isoforms [12].

In humans, Cx47 expression was detected in CNS and peripheral nervous system (PNS) [1], whereas mice express Cx47 (*Gjc2*; MGI: 2153060) only in oligodendrocytes contributing to gap junctional communication within panglial networks [13]–[15]. Mature oligodendrocytes in the CNS express Cx32 (*Gjb1*; MGI: 95719) and Cx29 (*Gjc3*; MGI: 2153041) in addition to Cx47 [16]. Astrocytes express Cx30 (*Gjb6*; MGI: 107588) and Cx43 (*Gja1*; MGI: 95713), while Cx26 (*Gjb2*; MGI: 95720) is expressed in some gray matter astrocytes [17]–[19]. Besides homotypic Cx47/Cx47 channels, heterotypic Cx47/Cx43 and Cx47/Cx30 channels showed functional coupling in cell culture experiments [11], [20].

Fracture replica immunogold labeling and immunohistological analyses suggested heterotypic channels between oligodendrocytes and astrocytes (O∶A) forming panglial networks [21], [22] and recent dye transfer experiments on oligodendrocytes in the corpus callosum confirmed O∶A intercellular communication. In addition, these experiments revealed functional interoligodendrocytic (O∶O) coupling. Both, O∶O and O∶A coupling were significantly affected in Cx47 deficient mice suggesting a crucial role for Cx47 gap junctions in panglial networks [23]. Accordingly, Cx47 deficient mice showed myelin vacuoles but behavioral or further anatomical abnormalities have not been observed as yet [13]. Mutations in the *GJB1* (MIM: 304040) gene coding for hCx32 protein result in the demyelinating peripheral neuropathy X-linked Charcot-Marie-Tooth disease (CMTX, MIM: 302800). Cx32 deficient mice showed only mild, late onset myelination deficits in the CNS [24] but additional deletion of Cx32 in Cx47 deficient mice results in severe myelin abnormalities and premature death. This indicates some functional redundance of these connexins expressed by oligodendrocytes.

Results from transfected cell cultures expressing mutant Cx47 proteins suggested that PMLD in humans is induced by either loss of Cx47 GJC function or hemichannel dysfunction [8], [25].

Since Cx47 null mice show only mild myelin abnormalities, in contrast to human PMLD patients, we generated a mouse model for PMLD by knock-in of the point mutation mCx47*M282T* into the endogenous Cx47 gene locus. The orthologous human missense mutation hCx47*M283T* was found homozygously in PMLD patients [1].

The Cx47*M282T* expressing mice enabled us to investigate whether loss of Cx47 function regarding O∶O and O∶A coupling, a transdominant effect on Cx32 or gain of detrimental function by intracellular effects of the mutant Cx47 contribute to the disease.

Young homozygous Cx47*M282T* expressing mice showed impaired motor coordination and balancing performance on the accelerating rotarod, decreased punctate Cx47 immunostaining and similar to Cx47 deficient mice, diminished O∶O coupling in the corpus callosum, compared to *wildtype* mice. Furthermore, like Cx47 deficient mice, homozygous Cx47*M282T* mice revealed retarded myelin formation during the first weeks after birth.

Similar to Cx47/Cx32 null mice, homozygous Cx47*M282T* mice deprived of Cx32 showed severe myelin abnormalities and died within the first four months after birth, demonstrating an essential function and partial redundancy of both connexin isoforms for maintenance of mouse myelin.

Our results indicate that loss of Cx47 function in GJC leads to myelin pathology in juvenile mice which can to a large extent be compensated during subsequent development to adulthood.

## Results

### Generation of Cx47*M282T* Mice

**Figure 1:**
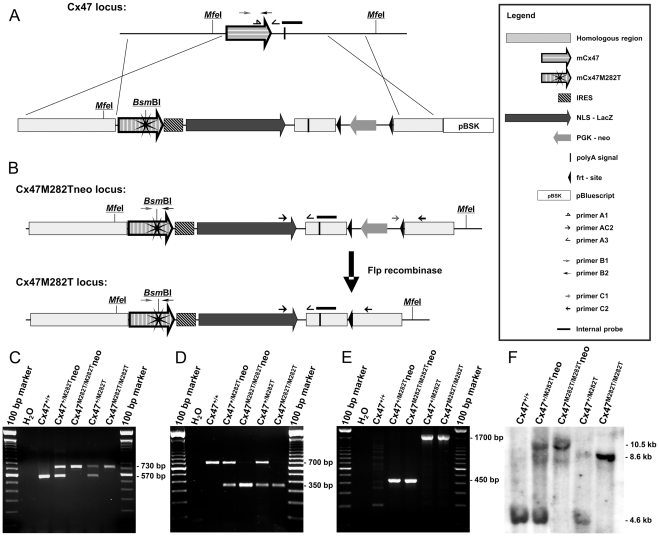
Generation of mCx47*M282T* mice with *LacZ* reporter gene. A, Scheme of homologous recombination of the targeting vector into the Cx47 coding region. The resulting transgenic allele (*Cx47M282Tneo*) includes mCx47*M282T* coding DNA, an internal ribosome entry site (IRES) followed by a nuclear localization signal (nls) fused to *LacZ* coding DNA and a neomycin selection cassette flanked by frt-sites. The endonuclease *Bsm*BI restriction site was generated by T to C transition on nucleotide 845 of the *Gjc2* coding region, resulting in the mutant mCx47*M282T*. B, Flp recombinase activity causes deletion of the neomycin selection cassette. Specific primer binding sites and *Mfe*I restriction sites are indicated. C, PCR products specific for wild-type (570 bp) and transgenic loci (730 bp) using DNA obtained from tail tip tissue. D, Presence of the mutant allele was proven by PCR amplification of a 700 bp Cx47 fragment and subsequent *Bsm*BI digestion. Only mutant alleles yielded the 350 bp fragment. E, PCR analysis resulted in amplification of the 1,700 bp fragment of the neomycin selection cassette deprived allele and the 450 bp fragment of the *Cx47M282Tneo* locus. F, Homologous recombination and different allelic combinations were confirmed by Southern blot hybridization using a probe derived from the 3′ region of Cx47. *Mfe*I digestion of genomic DNA prepared from liver resulted in the of 4.6 kb fragment for the Cx47 *wildtype* allele, the 10.5 kb fragment for the *Cx47M282Tneo* allele and the 8.5 kb fragment for the *Cx47M282T* allele.

The mCx47*M282T* mutation was generated by PCR mutagenesis using purified genomic DNA obtained from a C57BL/6 mouse as template. Codon 282 was mutated to cause a methionine residue to threonine transition by T to C exchange at nucleotide 845 of the mCx47 coding region which in addition resulted in a recognition site (5′-(N)_5_GAGACG-3′) of the restriction endonuclease *Bsm*BI. We replaced the mouse Cx47 coding region by the mutated mCx47*M282T* one, an internal ribosome entry site (IRES) followed by the *Escherichia coli* β-galactosidase (β-gal) coding DNA (*LacZ*), preceded by a nuclear localization sequence and a frt-site-flanked neomycin selection cassette by homologous recombination in HM-1 embryonic stem cells [26] (Figure 1A). Twenty-four of 377 neomycin resistant clones were positively characterized for homologous recombination by two individual PCRs. Homologous recombination was further verified by Southern blot analysis. Three of the targeted ES cell clones were microinjected into C57BL/6 blastocysts. Two clones yielded germline transmission chimeric mice that produced heterozygous *Cx47^+/M282T^neo* offspring after backcrossing with C57BL/6 mice. The frt-site-flanked neomycin resistance cassette was deleted by mating of *Cx47^+/M282T^neo* mice to *hACTB:FLPe* mice [27] which yielded the offspring *Cx47^+/M282T^* deprived of the neomycin selection cassette (Figure 1B). All mice further investigated harboured at least 96% of C57BL/6 genetic background. Mice carrying the mutant mCx47*M282T* allele heterozygously (*Cx47^+/M282T^* and *Cx47^+/M282T^neo*) were interbred to obtain *Cx47^+/+^*, *Cx47^+/M282T^* and *Cx47^M282T/M282T^* or *Cx47^+/+^*, *Cx47^+/M282T^neo* and *Cx47^M282T/M282T^neo* offspring, respectively. Genotypes were demonstrated by PCR (Figure 1C and E) and Southern Blot (Figure 1F) analysis. Genomic presence of the mutated mCx47*M282T* was analyzed by PCR and subsequent *Bsm*BI digestion (Figure 1D). Female as well as male *Cx47^+/M282T^* and *Cx47^M282T/M282T^* mice were fertile.

### m*Cx47M282T* and *LacZ* Reporter Gene Expression

**Figure 2:**
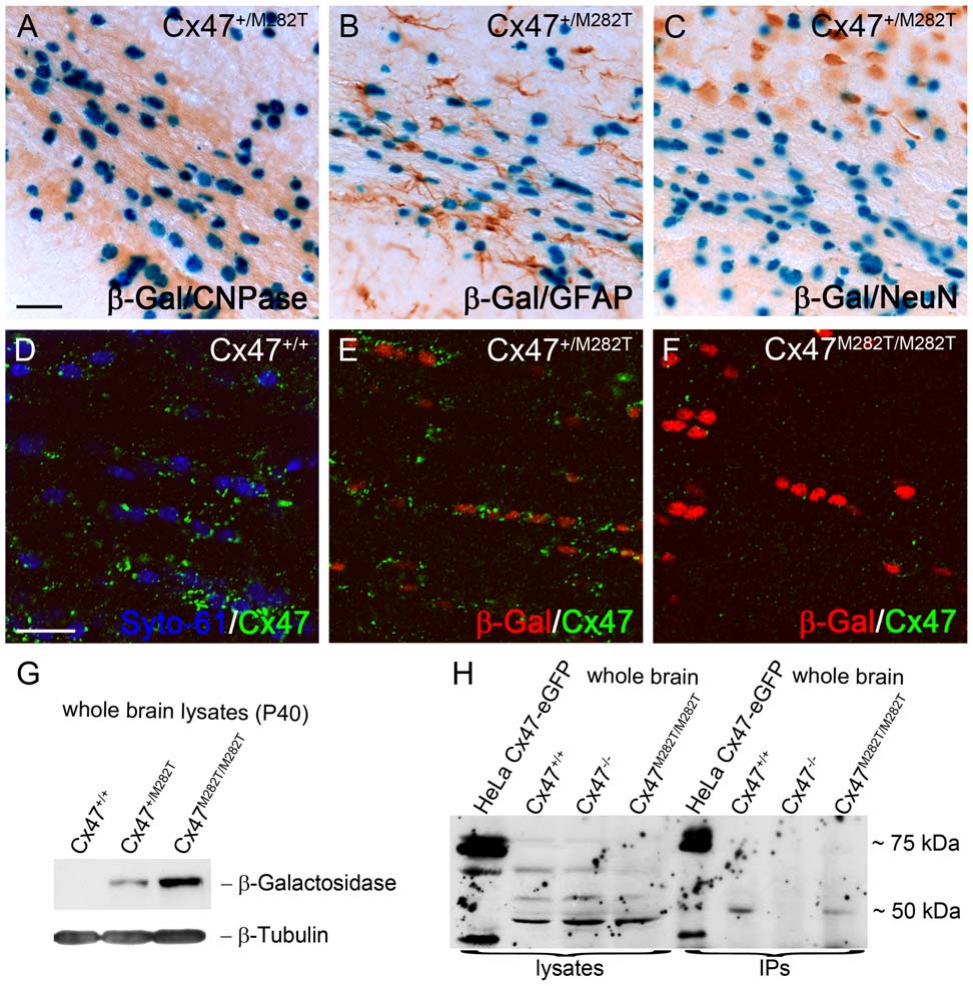
mCx47*M282T* and β-gal expression in oligodendrocytes of 40-day-old mutant mice. A–C, *LacZ* staining of 50 µm brain slices obtained from *Cx47^+/M282T^* mice. The β-gal activity is restricted to nuclear localisation. A, β-Gal positive cells in the corpus callosum show typical oligodendrocytic chain-like organization and coexpression of the oligodendrocytic marker CNPase. *LacZ* expression was not detectable in GFAP-positive astrocytes (B) or NeuN-positive neurons (C). D, Immunostaining with Cx47 antibodies (green) revealed signals in close proximity of Syto-61 stained nuclei in the corpus callosum of *Cx47^+/+^* mice. Weaker signals were also found more distal to the nuclei. E, Cx47 antibody stainings on heterozygous *Cx47^+/M282T^* mice resulted in weaker, but similarly localized immunosignals compared to those obtained on *wildtype* brain tissue. Cx47 gap junction immunosignals were predominantly localized close to β-gal positive nuclei indicated by antibody staining. F, Homozygous *Cx47^M282T/M282T^* mice showed apparent β-gal immunoreactivity, but robust Cx47 gap junction immunosignals were not detected in the perikarya. Very weak Cx47 signals were noticed in brain tissue of *Cx47^M282T/M282T^* mice. G, Immunoblot staining against β-gal on whole brain lysates of 40-day-old mice yielded strong signals with *Cx47^M282T/M282T^*, weaker signals with *Cx47^+/M282T^* and no signals with *Cx47^+/+^* tissue. H, Immunoblot analysis on Cx47 protein resulted in unspecific bands at 50 kDa of whole brain lysates obtained from *wildtype*, *Cx47^M282T/M282T^* and Cx47 deleted (*Cx47^−/−^*) tissue. Immunoprecipitated Cx47 was detected in *wildtype* and *Cx47^M282T/M282T^* tissue. Lysates of Cx47-eGFP fusion protein expressing HeLa cells yielded signals at 75 kDa as expected and additional weaker signals at approximately 40 kDa after immunoprecipitation of Cx47 and with crude HeLa cell lysate. Additional signals at 40 kDa in these lanes may represent the cleaved C-terminal region of Cx47. Scale bars: A–C, 50 µm; D–F, 20 µm.

Expression of the mutant mCx47*M282T* gene in transgenic animals resulted in a bicistronic mRNA coding for mCx47*M282T* and the reporter *LacZ* DNA. The β-gal encoded by *LacZ* DNA featured a nuclear localization signal and was detected by X-Gal and antibody staining. Translation of the *LacZ* mRNA was mediated by an IRES cassette. To further characterize *LacZ* expression, immunohistochemical analyses of cell type specific markers were combined with X-Gal staining. The reporter *LacZ* DNA was expressed in Cx47-positive cells (Figure 2E) and was coexpressed with the oligodendrocytic marker protein 2′,3′-cyclic nucleotide 3′ phosphodiesterase (CNPase) (Figure 2A). X-Gal staining was not colocalized with immunohistochemical signals of the neuronal marker (NeuN) or the astrocytic marker (GFAP) (Figure 2B and 2C). Immunofluorescence analyses showed a marked decrease of Cx47-positive puncta in *Cx47^M282T/M282T^* and, although less pronounced, in *Cx47^+/M282T^* brain slices compared to *wildtype*. Furthermore, Cx47 immunostaining revealed that the mCx47*M282T* protein does not exhibit the typical gap junctional immunosignals at oligodendrocytic somata [28]. β-Gal signals were obvious in *Cx47^+/M282T^* and *Cx47^M282T/M282T^* but absent in *wildtype* brain tissue (Figure 2D–2F). Immunoblot analysis of β-gal in brain lysates obtained from P40 mice yielded distinct signals in *Cx47^+/M282T^* and *Cx47^M282T/M282T^* around 120 kDa but not in corresponding *wildtype* controls (Figure 2G). Immunoprecipitation (IP) and subsequent immunoblot analysis against Cx47 revealed bands on *wildtype* and *Cx47^M282T/M282T^* brain lysates around 50 kDa. No signals were found in Cx47 deficient (*Cx47^−/−^*) control lysates after IP but immunoblot analysis on crude *Cx47^−/−^* whole brain lysates yielded false positive signals for all genotypes at approximately 50 kDa. To minimize precipitation and subsequent detection of cross reacting proteins, IPs and immunoblots were conducted using two distinct Cx47 antibodies (Zymed No. 37-4500 and Zymed No. 36-4700). HeLa cell control lysates yielded signals for the transfected Cx47-eGFP fusion protein around 75 kDa with crude lysates and after IP indicating accuracy of the procedure. Signals around 40 kDa in this lane are probably caused by the cleaved Cx47 C-terminus, since antibodies used were directed against peptides derived from the C-terminal region of Cx47.

### mCx47*M282T* Mice Show Characteristc Distribution Patterns of Oligodendrocytes during Postnatal Brain Development

**Figure 3:**
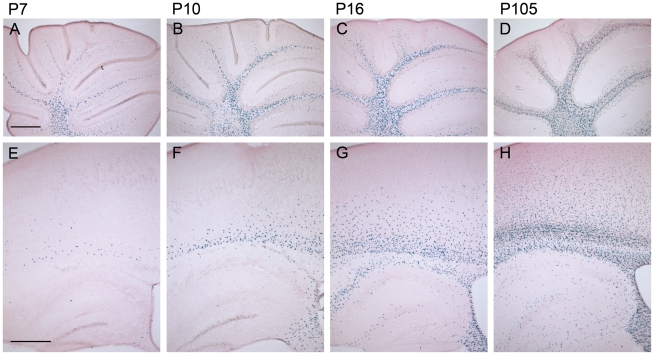
The number of β-gal positive cells increases during postnatal development of homozygous mCx47*M282T* expressing mice. Sagittal 50 µm brain sections were X-Gal stained for *LacZ* expression and counterstained with eosin. The *LacZ* reporter gene reflects the expression of Cx47. A, In the cerebellum of 7-day-old *Cx47^M282T/M282T^* mice several X-Gal stained oligodendrocytes were already found in the medullary centre. B, Stainings on cerebella of P10 mice revealed an increase in the number of β-gal positive cells in the white matter compared to P7 mice. Single β-gal positive cells were already found in the granular layer. C and D, Cx47 expressing cells further increased in number during development of the cerebellum. In addition to their localisation in the cerebellar white matter, *LacZ* expressing cells were found to be widespread among the granular layer in the cerebellum of 105 day old mice. E, Compared to the number of X-Gal stained cells in the white matter of P7 cerebellum, only few cells were stained in P7 cerebral white matter, localized in the corpus callosum and the hippocampal fimbria. Single cells were found in the cortex proximal to the corpus callosum. The hippocampal gray matter was almost free of β-gal positive cells at this developmental stage. F, On P10 the number of X-Gal stained cells was remarkably increased in the corpus callosum and the hippocampal fimbria compared to P7. β-Gal positive cells decreased in number from ventral to dorsal in the corpus callosum to the cortex. G, P16 animals showed robust *LacZ* expression in the white matter of the cerebrum including corpus callosum, hippocampal fimbria and hippocampal alveus. Furthermore, β-gal positive cells were present at the hippocampal fissure and near the hippocampal regions Cornu ammonis 1 (CA1) and CA3. Compared to P10 the number of *LacZ* expressing cells increased dramatically in cortical layers VI to IV of P16 mice. H, 105 days after birth Cx47 expressing cells represented by *LacZ* reporter gene stainings were localized all over the telencephalon with strong accumulation and typical oligodendrocytic chain-like organizaiton in the white matter. In gray matter, β-gal positive cells were scatterly distributed with a decreasing gradient ranging from layers VI to I of the neocortex. Scale bar: A–H, 500 µm.

An increase of β-gal positive cells in CNS white and gray matter of *Cx47^M282T/M282T^* mice (Figure 3) and *Cx47^+/M282T^* (not shown) was observed during postnatal development (P7–P105). At P7 β-gal positive cells were detected in the cerebellar white matter (Figure 3A), less in corpus callosum and in fimbria of hippocampus and only few isolated cells in the cerebral cortex (Figure 3E). At P10 the number of β-gal positive cells abundantly increased in the corpus callosum and the hippocampal fimbria. Unlike P7 mice, P10 animals displayed scattered β-gal positive cells localized in layer VI of the cerebral cortex (Figure 3F), whereas few β-gal positive cells were found in the cerebellar granular layer (Figure 3B). At P16 β-gal positive nuclei increased more than twofold in the cerebellar white matter and granular layer compared to P10 mice (Figure 3B and C). At this developmental stage, we also observed β-gal positive cells in the hippocampus, predominantly localized at the hippocampal fissure and alveus, and in layers VI-IV of the cerebral cortex, while the number of *LacZ* expressing cells in white matter tracts was further increased (Figure 3G). After 3.5 months of postnatal development (P105) the number of β-gal positive cells was increased in white matter and adjacent gray matter compared to P16 mice (Figure 3D). X-Gal stained nuclei were furthermore present in all layers of the cerebral cortex, decreasing in number from layer VI to layer I. Isolated β-gal positive cells were found widespread over the hippocampal gray matter (Figure 3H). In cerebellar and cerebral white matter of P105 mice *LacZ* expressing cells were often organized in a chain-like structure parallel to the direction of the fibers, as typically observed for oligodendrocytes in white matter (Figure 3D and 3H).

### Neuropathologic Abnormalities in Juvenile *Cx47^M282T/M282T^* Mice

**Figure 4:**
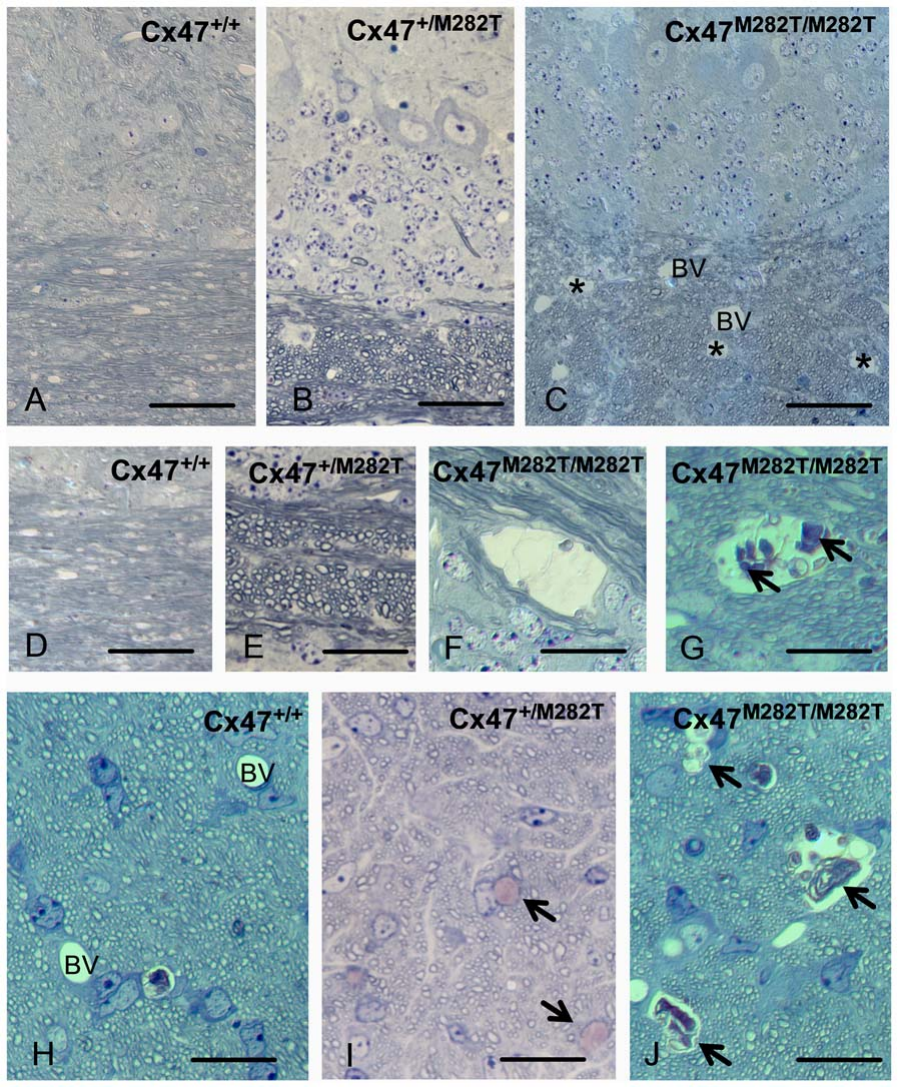
Toluidine blue/pyronin g stained semi-thin sections reveal cystic spaces in white matter of *Cx47^M282T/M282T^* mice. Semi-thin sections of cerebellar white matter of *Cx47^+/+^* (A, C) *Cx47^+/M282T^* (B, E, I) and *Cx47^M282T/M282T^* (C, F, G, J) mice are shown after staining with toluidine blue/pyronin g. Cystic spaces in white matter are indicated by asterisks in C and can be delineated from blood vessels (BV) due to their irregular lining and absence of endothelia. At higher magnification (F, G) cysts can be shown to contain cellular debris (arrows in G). No alterations were seen in cerebella from heterozygous mice (B, E). In the prechiasmatic optic fascicle of *Cx47^M282T/M282T^* mice, similar cysts are evident (arrows in J, compared to blood vessles (BV) in the WT control shown in H). Scale bars: A–C, 50 µm; D–J, 20 µm.

Sixteen day-old *Cx47^M282T/M282T^* mice featured a neuropathologic phenotype affecting mainly the white matter in some regions of the CNS (Figure 4). Within the cerebellar white matter, cystic spaces of about 100 µm diameter were found, filled with cellular debris, sometimes featuring an onion shell – like lamellar pattern. Within the cerebellar gray matter, scattered groups of mainly Purkinje neurons could be seen at different stages of degeneration.

A similar cystic degeneration of CNS white matter was observed in the prechiasmatic optic fascicle featuring about 2–3 cysts of about 100 µm diameter per cross section.

Interestingly, while the effects in cerebellum and optic fascicle were rather constant between different experimental animals, the effects of mCx47*M282T* expression in the remainder of the telencephalon were much more variable. Most notably, the main white matter tracts like fimbria hippocampi, corpus callosum or anterior commissure did not show any regularly occurring cystic degeneration. Within the gray matter, we inconstantly observed groups of degenerating cells in the hippocampal field CA1 and in the subgranular zone of the dentate gyrus (Figure S1A and S1B).

Next to the neuronal damage, we inconstantly observed cell debris and degenerating small cells at the outer aspect of numerous brain capillaries, indicative of a disseminated, and again highly variable destruction of perivascular astroglia (Figure S1C–S1E).

In heterozygous mice, the morphology of the cerebellum was essentially normal (Figure 4B, E): likewise, lesions within the optic fascicle were almost absent except for a small number (about 2 per cross section of one fascicle) of individual cells being swollen and degenerated (Figure 4I).

### Behavioral Analysis

We investigated general activity, motor coordination and motor learning performance in juvenile (23 days old) and adult (three months old) mCx47*M282T* mice. Since PMLD is caused by an autosomal recessive mutation we also investigated whether heterozygous mice would exhibit an intermediate phenotype ranging between homozygous and wild-type mice. For this purpose pre-planned pair-wise comparisons between wild-type mice and homozygous as well as between wild-type mice and heterozygous mice were performed.

#### Open-field behavior of juvenile mice

**Figure 5:**
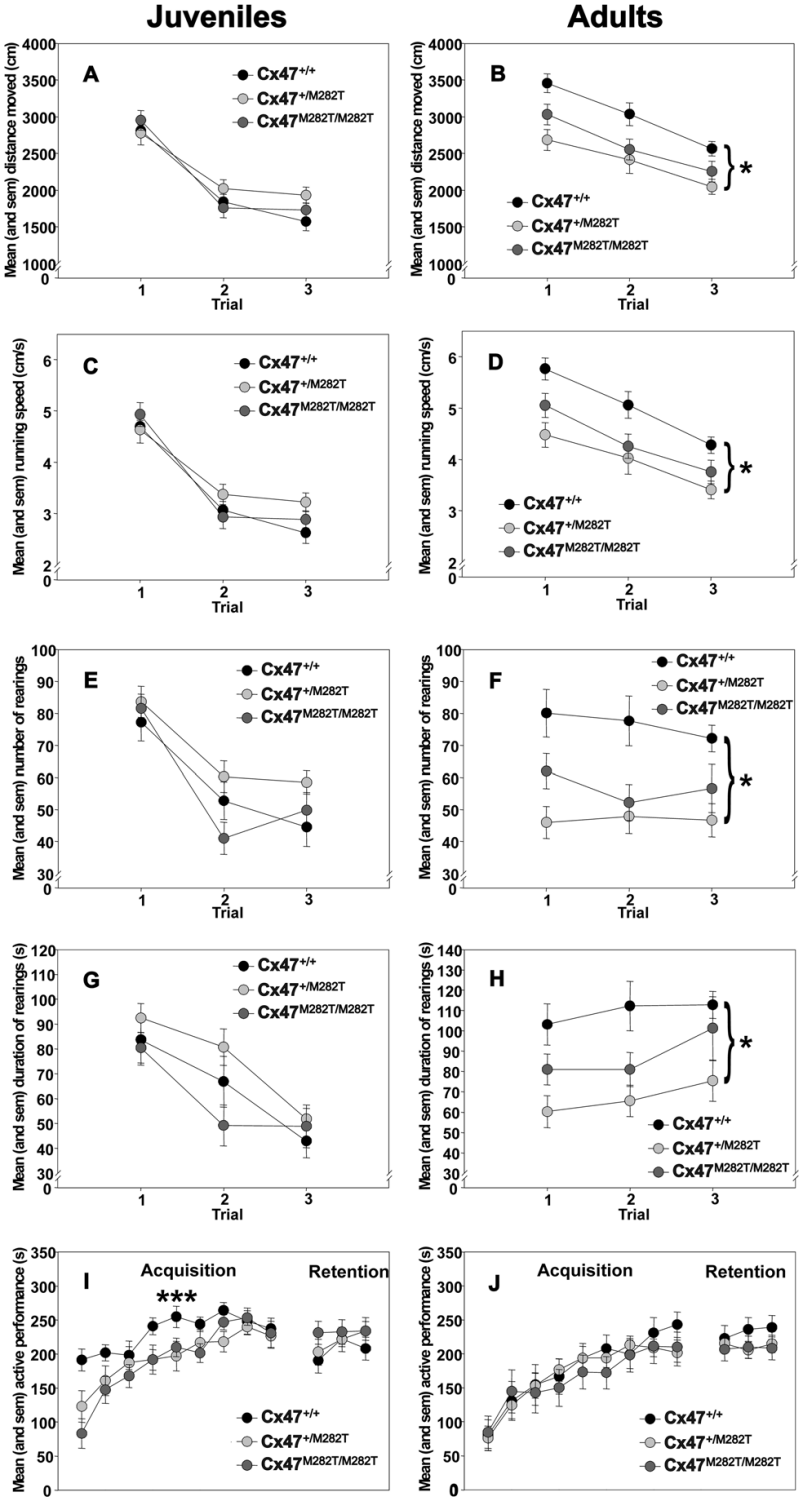
Open-field behavior and rotarod performance of juvenile (23 days) and adult (3 months) *Cx47^M282T/M282T^*, *Cx47^+/M282T^*, and *Cx47^+/+^* mice. A and B, Circles represent mean ± sem locomotion (distance moved in cm) on indicated trials. C and D, Circles represent mean ± sem running speed (in cm/s) on indicated trials. E and F, Circles represent mean ± sem number of rearings on indicated trials. G and H, Circles represent mean ± sem duration of rearings (in s) on indicated trials. I and J, Motor coordination and balancing on the accelerating rotarod. Circles represent mean ± sem time of active performance (in s) on acquisition and retention trials. ***: *Cx47^+/+^* versus *Cx47^M282T/M282T^*, p = 0.01; Bonferroni test. *: *Cx47^+/+^* versus *Cx47^+/M282T^*, p = 0.01; Bonferroni test.

Across the three trials in the open-field, the animals, irrespective of genotype, showed significant reductions in the distance moved (main effect of *trials*: p<0.001; repeated measures ANOVA; Figure 5A), running speed (p<0.001; Figure 5C), as well as in the number (p<0.001; Figure 5E) and duration of rearings (p<0.001; Figure 5G). However, there were no significant main effects of *genotype* for the variables distance moved, running speed, or the number and duration of rearings (all p's>0.05). Except for the distance moved parameter (*genotype×trial* interaction: p = 0.036), the *genotype × trial* interactions for the remaining parameters were all not significant (all p's>0.05). Interestingly, the analysis of the rearing behavior (number and duration) during the second trial yielded an unexpected genotype distribution. On the second trial, the Cx47*^+/+^* mice exhibited an intermediate level of rearing activity, while the Cx47*^+/M282T^* showed higher and the Cx47*^M282T/M282T^* mice the lower rearing activity as compared to controls. Indeed, Student t-test comparisons between Cx47*^+/M282T^* and Cx47*^M282T/M282T^* mice for single trials revealed a significant difference in the number (p = 0.02) and duration of rearings (p = 0.0154) during trial 2 but not during the trials 1 and 3. There were no significant differences in terms of rearing behavior on trials 1–3 between the Cx47*^M282T/M282T^* or Cx47*^+/M282T^* and the Cx47*^+/+^* mice (all p's>0.05). In repeated (ANOVA treated) measurements of the behavioral parameters, no main effects of genotype were detected. These results suggest that expression of the *mCx47M282T* mutation has no detrimental effects on open-field behavior in juvenile mice.

#### Open-field behavior of adult mice

Across the three exposures to the open-field, the animals showed significant decrements in the distance moved (main effect of *trials*: p<0.001; repeated measures ANOVA; Figure 5B), running speed (p<0.001; Figure 5D), and duration of rearings (p = 0.042; Figure 5H), but not in the number of rearings (p>0.05; Figure 5F). The open-field behavior of the three groups was significantly different. The groups differed in terms of the distance moved (main effect of *genotype*: p = 0.002; repeated measures ANOVA; Figure 5B), running speed (p = 0.002; Figure 5D), as well as regarding the number (p = 0.001; Figure 5F) and duration (p = 0.003; Figure 5H) of rearings. Post-hoc pair-wise comparisons revealed that compared to *Cx47^+/+^* control mice, the *Cx47^+/M282T^* mice showed significantly reduced locomotion (p = 0.002; Bonferroni test), running speed (p = 0.002) and rearing activity (number: p = 0.001; duration: p = 0.002). The *Cx47^+/+^* mice also showed higher levels of open-field activity relative to the *Cx47^M282T/M282T^*. However, these differences failed to reach the predetermined level of statistical significance (p's>0.05). These results suggest that the heterozygous expression of the mCx47*M282T* mutation in the adult mouse impairs open-field behavior.

#### Motor coordination and balancing performance of juvenile mice

The animals performance improved across the nine acquisition trials (main effect of trials; p<0.001; repeated measures ANOVA, Figure 5I). There were no significant main effects of *genotype* (p = 0.097) or *genotype×trial* interaction (p = 0.093). However, the p-values indicated trends towards significance. Pre-planned pair-wise comparisons revealed that compared to *Cx47^+/+^* control mice, *Cx47^M282T/M282T^* mice showed significantly impaired rotarod performance during the acquisition stage of the task (main effects of *genotype*: p = 0.016). *Cx47^+/+^* mice also outperformed heterozygous *Cx47^+/M282T^* mice. However, this difference failed to reach the predetermined level of statistical significance (p>0.05). In contrast, there were no significant differences between groups during the retention test (p's>0.05). These results suggest that the homozygous expression of the mCx47*M282T* mutation in juvenile mice has a detrimental effect on the acquisition of the rotarod task.

#### Motor coordination and balancing performance of adult mice

The animals performance improved across the nine acquisition trials (main effect of trials; P<0.001; repeated measures ANOVA, Figure 5J). However, there were no significant main effects of *genotype* or *genotype×trial* interaction (p's>0.05). Similarly there were no significant main effects of *genotype* or *genotype × trial* interaction or pre-planned pair-wise comparisons (p's>0.05) during the retention phase. Pre-planned pair-wise comparisons between Cx47^+/+^ control and Cx47^M282T/M282T^ as well as between Cx47^+/+^ control Cx47^+/M282T^ mice revealed no significant differences (p's>0.05). These results show that the mCx47*M282T* mutation has no effect on rotarod performance in the adult mouse.

Taken together, three-week-old homozygous Cx47*M282T* expressing mice showed impaired motor coordination and balancing performance on the accelerating rotarod. Three-month-old homozygous Cx47*M282T* mice had compensated the rotarod impairment caused by mutant Cx47 expression. Compared to *wildtype* controls, three-week-old homozygous and heterozygous Cx47*M282T* expressing mice showed normal behavior in the open-field, while three-month-old heterozygous Cx47*M282T* mice exhibited reduced motor activity in the open-field.

### mCx47M282T Mutation Reduces Oligodendrocyte Coupling

**Figure 6:**
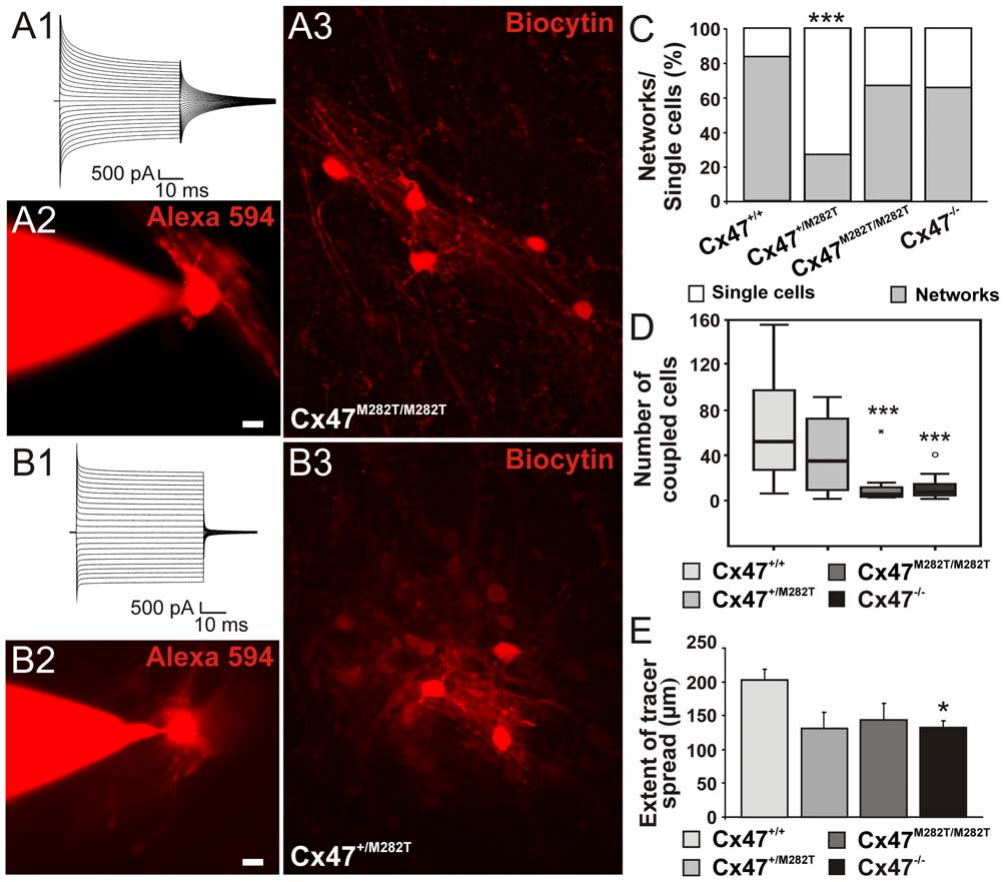
Comparison of oligodendrocytic coupling in the corpus callosum of postnatal 10- to 15-day-old *Cx47^+/M282T^*, *Cx47^M282T/M282T^*, and *Cx47^−/−^* compared to *Cx47^+/+^* mice[**23**]. A1 and B1, Membrane currents of oligodendrocytes were recorded in response to a series of voltage steps ranging from −170 and + 50 mV (50 ms, 10 mV increments) from a holding potential of −70 mV. A2 and B2, Oligodendrocytes were identified by their typical morphology as revealed by dialysis with the fluorescent dye Alexa 594. A3 and B3, Superpositions of sequential confocal stacks indicating biocytin/streptavidin-Cy3 labeled networks. C–E, Quantification of networks in homozygous and heterozygous mCx47*M282T* as well as *Cx47^−/−^* mice compared to *wildtype* animals. C, Percentage of injected cells which were coupled to at least one other cell and thereby formed a network (dark gray) versus injected uncoupled cells (light gray). Note that in Cx47^+/M282T^ mice only 27% of injected oligodendrocytes were found to be coupled. Asterisks indicate statistical significance (Chi Square Test, *** p<0.001). D, Boxplot indicating median (dark line), 25^th^ and 75^th^ percentiles (upper and lower hinges, respectively) of the number of coupled cells per network in Cx47^M282T/M282T^ and Cx47^+/M282T^ compared to wildtype mice. Similarly to *Cx47^−/−^* mice, Cx47^M282T/M282T^ animals displayed a significant reduction in the number of cells participating in the oligodendrocytic network (6%; 4%–13%; Kruskal-Wallis test, p = 0.001, Mann-Whitney U-test, *** p<0.001). E, Extent of tracer spread as defined by the largest distance between two somata within the coupled network. No significant difference was observed between homozygous and heterozygous *mCx47M282T* mice compared to wildtype animals. In *Cx47^−/−^* mice, the extent of tracer spread was significantly reduced compared to *wildtype* animals (132±11 average, One way ANOVA p<0.05, Bonferroni post hoc test, p<0.05).

To address the question whether homozygous and heterozygous expression of mCx47M282T mutation affects coupling, dye transfer experiments were performed in the corpus callosum of acute coronal slices obtained from P10–15 mice. In each slice, single oligodendrocytes were dialyzed by whole cell patch-clamp with the gap junction permeable tracer biocytin, as previously described [23]. Membrane currents of the injected oligodendrocyte were recorded in response to a series of hyperpolarizing and depolarizing voltage steps (10 mV increment) from −170 mV to +50 mV for 50 ms (Figure 6A1, 6B1). The cohort of *wildtype* and *Cx47^−/−^* control mice was the same as previously published, since the experiments reported here were done in parallel with the former study [23].

**Figure 7:**
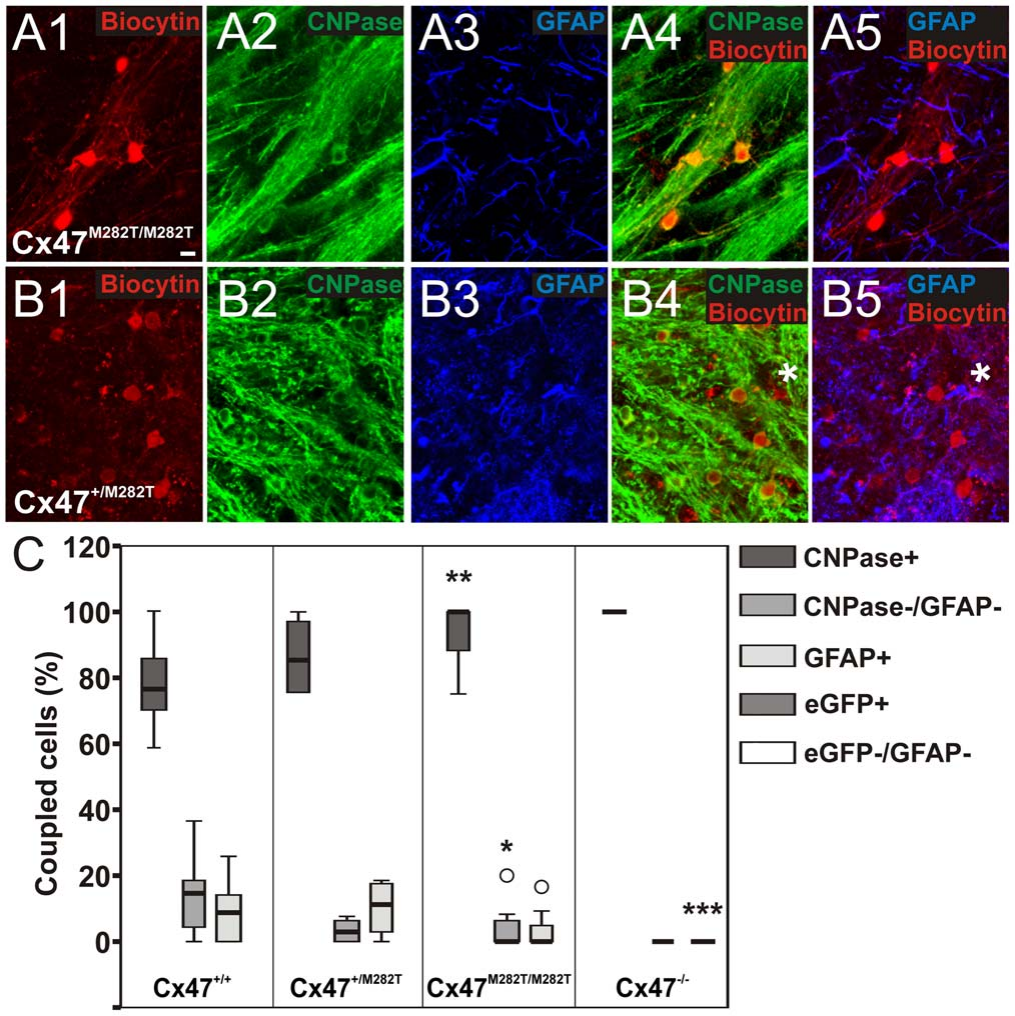
Identification of glial cell-types within networks of *Cx47^+/M282T^*, *Cx47^M282T/M282T^*, and *Cx47^−/−^* mice (P10–P15) in comparison to Cx47^+/+^ animals of the same age [**23**]. A1–A5, Example of cell-type identification in biocytin labeled networks of *Cx47^M282T/M282T^* mice. Biocytin was detected with streptavidin-Cy3 (A1) and oligodendrocytes were identified by CNPase immunostaining (A2). A3, Immunolabeling of the same section for the astrocytic marker GFAP. A4, Overlay of A1 and A2. A5, Overlay of A1 and A3. In this network no coupled cell expressed GFAP. B1–B5, Biocytin labeling with streptavidin-Cy3 (B1) combined with immunostaining for CNPase (B2) and GFAP (B3) in the corpus callosum of Cx47^+/M282T^ mice. B4, Overlay of B1 and B2. B5, Overlay of B1 and B3. Note the GFAP-positive astrocyte (asterisk) labeled with biocytin. The same cell was CNPase-negative (B4, asterisk). C, Boxplot indicating median (dark line), 25^th^ and 75^th^ percentiles (upper and lower hinges, respectively) of the population of cells coupled within networks. Values of specific glial cell types are given as percentage of the number of biocytin-positve cells per network. In both *Cx47^M282T/M282T^* and *Cx47^+/M282T^* mice the majority of coupled cells were CNPase-positive, a small population lacked immunopositive signals for both CNPase and GFAP, and few GFAP-positive cells were found. *Cx47^+/M282T^* expression in comparison to wildtype tissue did not result in significant differences regarding the population of coupled cells per network. In *Cx47^M282T/M282T^* mice, immunostaining revealed an increased amount of CNPase-positive cells in the panglial networks (100%; 86%–100%; Kruskal-Wallis test, p<0.01, Mann-Whitney U-test, **p<0.01). A significantly diminished number of cells negative for both CNPase and GFAP were detected in these networks (0%; 0%–7%; Kruskal-Wallis test, p<0.01, Mann-Whitney U-test, *p<0.05). In Cx47^−/−^ mice all coupled cells were identified as eGFP-positive, i.e. oligodendrocyes [23]. No significant difference in oligodendrocyte-to-oligodendrocyte or oligodendrocyte-to-astrocytel coupling between *Cx47^M282T/M282T^* and Cx47^+/M282T^ mice was observed.

We distinguished between oligodendrocytes participating in glial networks and uncoupled oligodendrocytes by biocytin injection into oligodendrocytes and subsequent streptavidin-Cy3 labeling (Figure 6C). The number of coupled cells and extent of tracer spread (µm) were determined for the networks identified (Figure 6D, 6E) and glial cells whithin these networks were further characterized by marker protein costainings (Figure 7). For a detailed numeric representation see Table 1.

**Table 1:** Quantification of dye coupling in *Cx47^+/M282T^*, *Cx47^M282T/M282T^*, and *Cx47* ^−/−^ mice in comparison to *Cx47^+/+^* at P10–P15.

	*Cx47^+/+^*	*Cx47^+/M282T^*	*Cx47^M282T/M282T^*	*Cx47^−/−^*
**Networks of coupled cells (%)**	83 *(n = 24)*	27 (n = 15)***	67 *(n = 15)*	65 *(n = 20)*
**Extent of tracer spread (µm)**	203±16	131±24	143±27	132±11*
**Number of coupled cells per network**	52 (27–98)	36 (6–82)	6 (4–13)***	8 (4.5–18) ***
**CNPase+ cells per network (%)**	76 (70–85)	85 (76–99)	100 (86–100)**	-
**CNPase−/GFAP− cells per network (%)**	16 (5–20)	3 (0–7)	0 (0–7)*	-
**GFAP+ cells per network (%)**	9 (0–15)	11 (1–18)	0 (0–6)	0 (0–0) ***
**eGFP+ cells per network (%)**	-	-	-	100 (100–100)
**eGFP−/GFAP− cells per networks (%)**	-	-	-	0 (0–0)

Networks are shown as percentage of biocytin-injected oligodendrocytes found coupled to at least one adjacent cell. *** p<0.001 *Cx47^+/M282T^* versus *Cx47^+/+^*; Chi Square Test. Data regarding the extent of tracer spread are reported as average ± SEM; * p<0.05 *Cx47^−/−^* versus *Cx47^+/+^*; One way ANOVA followed by Bonferroni post hoc test, p<0.05. Quantification of the number of coupled cells is indicated as median (25^th^–75^th^ percentiles). *** p<0.001 *Cx47^M282T/M282T^* versus *Cx47^+/+^*; Mann-Whitney U-test, Kruskal-Wallis test, p = 0.001. *** p<0.001 *Cx47^−/−^* versus *Cx47^+/+^*; Mann-Whitney U-test, Kruskall-Wallis test, p<0.001. Quantification of the population of coupled cells (CNPase+, CNPase−/GFAP−, GFAP+, eGFP+, eGFP−/GFAP−) is indicated as median (25^th^–75^th^ percentiles). ** p<0.01 *Cx47^M282T/M282T^* versus *Cx47^+/+^*; Mann-Whitney U-test, Kruskal-Wallis test, p<0.01. * p<0.05 *Cx47^M282T/M282T^* versus *Cx47^+/+^*; Mann-Whitney U-test, Kruskal-Wallis test, p<0.01. *** p<0.001 *Cx47^−/−^* versus *Cx47^+/+^*; Mann-Whitney U-test, Kruskall-Wallis test, p<0.001. *(n)*, number of experiments.

In Cx47^M282T/M282T^ mice, 67% of the single oligodendrocytes dialyzed with biocytin formed networks (10 out of 15 slices from 3 mice). In these ten networks the median value of biocytin-positive (coupled) cells was 6 (4–13; values indicate 25^th^–75^th^ percentiles, respectively), with a significant reduction by 88% in the number of coupled cells per network as compared to wildtype mice (Kruskal-Wallis, p = 0.001, Mann-Whitney U-test, p<0.001). Measuring the largest distance between two cells within a network indicated that the mean extent of tracer spread for Cx47^M282T/M282T^ mice compared to wildtype animals was not significantly different (Figure 6C–6E).

To determine whether homozygous mCx47M282T expression affected coupling of oligodendrocytes to neighboring oligodendrocytes and astrocytes, biocytin/streptavidin-Cy3 labelling was combined with immunostaining for the oligodendrocytic marker CNPase and the astrocytic marker GFAP. In Cx47^M282T/M282T^ mice the majority of biocytin-positive cells coupled within a given network were CNPase-positive (100%; 86%–100%), with a significant increase in comparison to wildtype (see table 1; Kruskal-Wallis, p<0.01, Mann-Whitney U-test, p<0.01) (Figure 7A and 7C). GFAP-positive cells colabeled with biocytin/streptavidin-Cy3 (0%; 0%–6%) were found only in 3 out of ten networks (Figure 7A and C). However, the observed reduction was not significant compared to wildtype mice (see Table 1). Within a network analyzed we detected a population of CNPase- and GFAP-negative cells, which was significantly reduced to a median value of 0% (0%–7%) compared to wildtype animals (see table 1; Kruskal-Wallis p<0.05, Mann-Whitney U-test, p<0.01) (Figure 7A and 7C).

In heterozygous Cx47^+/M282T^ mice (Figure 6B1–6B3) only 4 out of 15 biocytin injections into single oligodendrocytes revealed intercellular coupling (27% formed networks, 4 mice), with networks consisting of a median value of 36 cells (6–82) and tracer spreading up to 193 µm (average 131±24 µm) (Figure 6). The observed decrease in the number of oligodendrocytes forming networks was significantly different in comparison to wildtype mice (see table 1; Chi-Square Test, Chi Square = 12.523, p<0.001) [23]. However, the number of coupled cells within a given panglial network and the extent of biocytin spread were not significantly affected as compared to wildtype animals [23]. Similar to the networks detected in brain slices obtained from wildtype mice, the majority of biocytin-positive cells were CNPase-positive, 3% (0%–7%) coupled cells were CNPase- and GFAP-negative, while the remaining 11% (1%–18%) were GFAP-positive astrocytes (Figure 7B and 7C).

Thus, homozygous mCx47M282T expression resulted in a decrease in the number of coupled cells within the network of cells coupled to the injected oligodendrocyte. No significant difference in number of coupled cells between Cx47^M282T/M282T^ and Cx47^−/−^ mice was observed. Heterotypic coupling of oligodendrocytes to GFAP-positive astrocytes was impaired but not completely abolished and coupling to the population of CNPase- and GFAP-negative cells was significantly affected. Heterozygous mCx47M282T expression caused a decrease by 68% in the number of oligodendrocytes forming networks in comparison to wildtype animals, but did not impair the number of coupled cells within a given network. However, in Cx47^+/M282T^ mice neither the number of coupled cells within a network nor coupling of oligodendrocytes to astrocytes and to the subpopulation of CNPase- and GFAP-negative cells was significantly affected.

### Developing Cerebella of *Cx47^M282T/M282T^*, *Cx47^+/M282T^*, and *Cx47 null* Mice Show Retarded Myelin Formation, Astrogliosis, As Well As Increased Iba1 Expression

**Figure 8:**
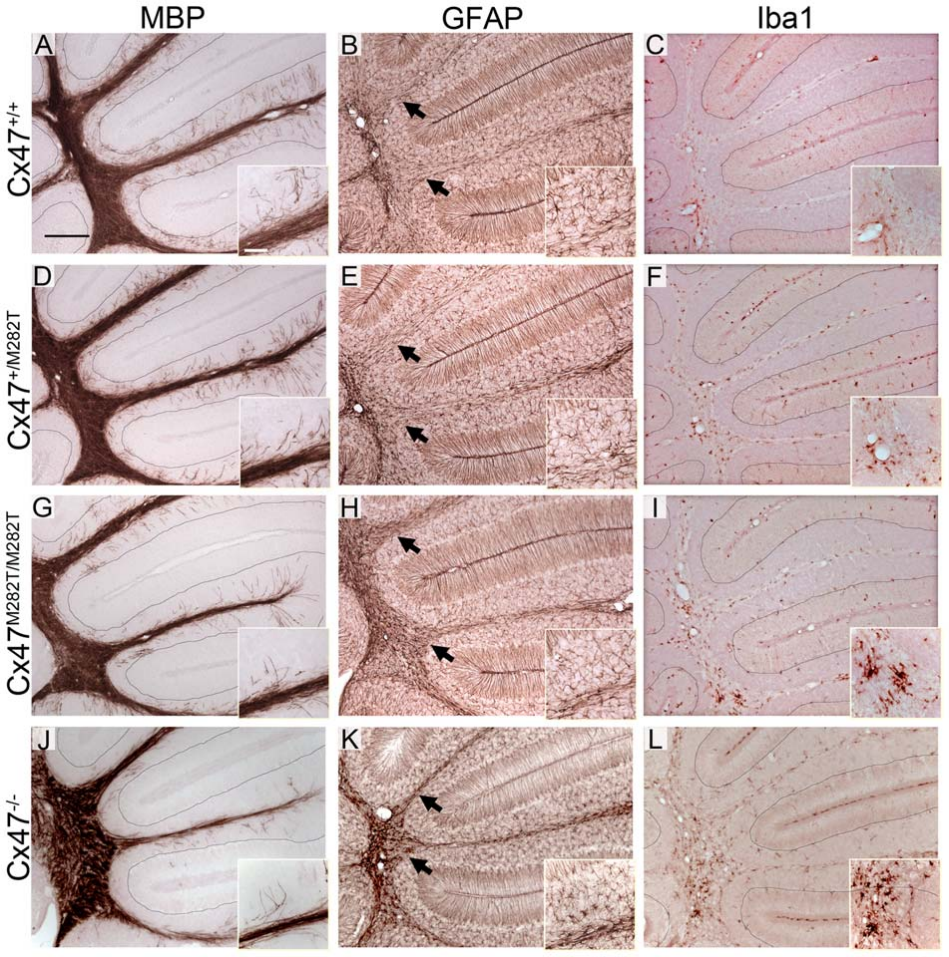
Deletion and mutation of Cx47 results in disturbed MBP expression accompanied by astrogliosis and microglial activation in juvenile mice. A, D, G and J, Immunohistochemical analysis on 25 µm sagittal brain slices obtained from 10-day-old animals revealed fewer myelinated, MBP-positive fine fibers pervading the cerebellar granular layer of *Cx47^+/M282T^*, *Cx47^M282T/M282T^* and *Cx47^−/−^* mice compared to *Cx47^+/+^* littermates. B, E, H and K, Delayed myelin formation is accompanied by elevated GFAP expression, indicating astrogliosis in the cerebellar white matter which appeared minor in *Cx47^+/M282T^* tissue but was pronounced in cerebella of P10 *Cx47^M282T/M282T^* and *Cx47^−/−^* mice. Arrows indicate the transition of white matter to the granule cell layer. Compared to *wildtype* tissue fewer GFAP-positive cells were localized in the granular layer of *Cx47^M282T/M282^* cerebella. Note the GFAP-positive radial processes of Bergmann glia cells pervading the molecular layer. Signals of GFAP-positive Begmann glia processes were used as internal control for staining intensities. C, F, I and L, Microglia were constantly distributed throughout the whole brain. Activated microglia displayed increased levels of Iba1. In P10 cerebella microglia with elevated Iba1 expression were found to a greater extent in brain slices obtained from *Cx47^+/M282T^*, *Cx47^M282T/M282T^* and *Cx47^−/−^* animals relative to *Cx47^+/+^* tissue. Auxiliary lines (MBP and Iba1 stainings) indicate the granular and Purkinje layer transition. Scale bars: A–L, 200 µm; insets, 50 µm.

**Figure 9:**
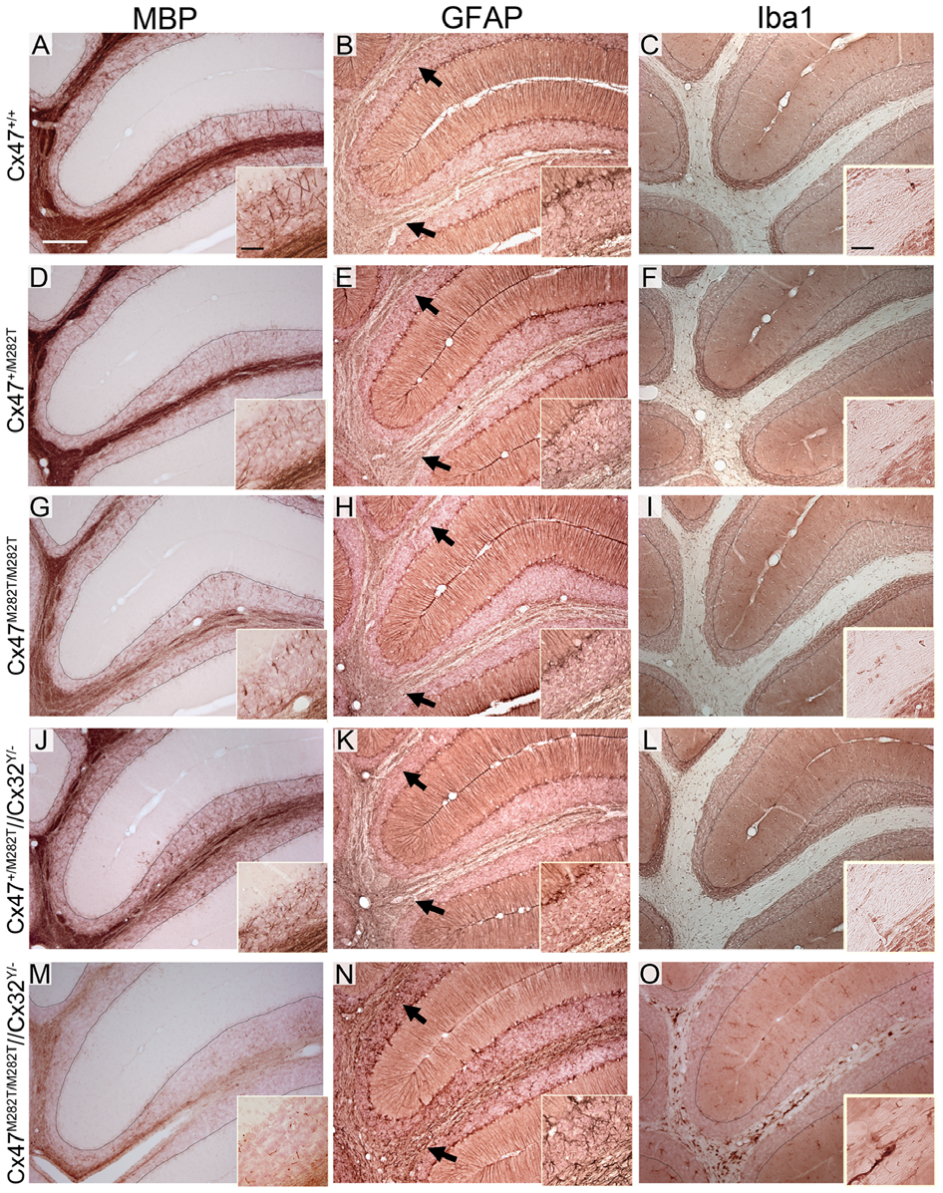
Insufficient myelination resulting from loss of Cx47 function is largely, although not completely, compensated in adult *Cx47^+/M282T^* and *Cx47^M282T/M282T^* mice. Additional deletion of Cx32 in homozygous mCx47*M282T* expressing mice resulted in severe myelin malformation, astrogliosis and microglia activation. A, Immunohistochemical analysis on 25 µm sagittal cerebellar slices obtained from 90-day-old animals yielded strong myelin staining in the granular layer of *wildtype* mice. D, G and J, Only few MBP-positive fibers attaining the Purkinje cell layer were found in corresponding regions of *Cx47^+/M282T^*, *Cx47^M282T/M282T^* and *Cx47^+/M282T^/Cx32^Y/−^* mice. M, MBP immunohistochemical signals were very weak in *Cx47^M282T/M282T^/Cx32^Y/−^* animals and appeared like debris in the granular layer. B, E, H, K and N, GFAP stainig on cerebellar slices of all five genotypes revealed astrogliosis only in *Cx47^M282T/M282T^/Cx32^Y/−^* animals (N) whereas *Cx47^+/+^*, *Cx47^+/M282T^*, *Cx47^M282T/M282T^* and *Cx47^+/M282T^/Cx32^Y/−^* mice did not display increased GFAP expression in the cerebellar white matter. H inset, Like P10 mice (Figure 8H), adult *Cx47^M282T/M282T^* mice harboured fewer GFAP-positive cells in the granular layer compared to *Cx47^+/+^* and *Cx47^+/M282T^* mice. Arrows indicate the transition of white matter to the granule cell layer. Signals of GFAP-positive Begmann glia processes were used as internal control for staining intensities. O, Furthermore, considerable numbers of Iba1 expressing activated microglial cells were present in *Cx47^M282T/M282T^/Cx32^Y/−^* cerebellar white matter. All other genotypes investigated did not display microglia with increased Iba1 exression in cerebellar tissue. Auxiliary lines indicate the granular and Purkinje cell layer transition. Scale bars: A–O, 200 µm; insets MBP and GFAP stainings, 50 µm; insets Iba1 stainings, 25 µm.

In cerebellar slices obtained from P10 *Cx47^M282T/M282T^* and *Cx47^−/−^* mice vacuoles were occasionally present in the white matter tract and MBP stainings appeared inhomogeneous compared to *wildtype* and cerebella of *Cx47^+/M282T^* mice. Furthermore, *Cx47^M282T/M282T^* and *Cx47^−/−^* mice showed fewer fine fibers in the granular layer than *wildtype* littermates and these fibers did not reach the Purkinje cell layer in *Cx47^+/M282T^*, *Cx47^M282T/M282T^* and *Cx47^−/−^* as observed in *Cx47^+/+^* slices (Figure 8A, 8D, 8G and 8J). Expression of the mCx47*M282T* protein resulted in conspicuous astrogliosis in the cerebellar white matter of *Cx47^M282T/M282T^* and *Cx47^−/−^* animals as indicated by up-regulation of GFAP expression, while *Cx47^+/M282T^* mice displayed only a mild increase in GFAP levels within white matter tracts (arrows). Furthermore, fewer GFAP-positive cells were visible in the cerebellar granular layer of *Cx47^M282T/M282T^* mice (Figure 8B, 8E, 8H and 8K). Elevated ionized calcium-binding adapter molecule 1 (Iba1) expression indicated accumulation/activation of microglial cells in the cerebellar white matter of *Cx47^+/M282T^*, *Cx47^M282T/M282T^* and *Cx47^−/−^* animals (Figure 8C, F, I and L). However, astrogliosis and activation of microglial cells could not be detected any longer in P90 *Cx47^+/M282T^* and *Cx47^M282T/M282T^* mice (Figure 9E, H, F and I). In the cerebellar granular layer of 3-month-old *Cx47^M282T/M282T^* mice, GFAP-positive cells were still diminished compared to corresponding *Cx47^+/M282T^* and *Cx47^+/+^* mice (Figure 9B, F and H). MBP staining on cerebella slices of adult *Cx47^M282T/M282T^* mice revealed uneven signals in the white matter compared to *wildtype* littermates. Vacuoles could not be detected anymore but the fine myelin fibers pervading the granular layer were almost absent in cerebella of *Cx47^+/M282T^* and *Cx47^M282T/M282T^* mice (Figure 9A, 9D and 9G).

### Oligodendrocytic Myelin Protein Synthesis Is Impaired in mCx47*M282T*-Expressing Mice

**Figure 10:**
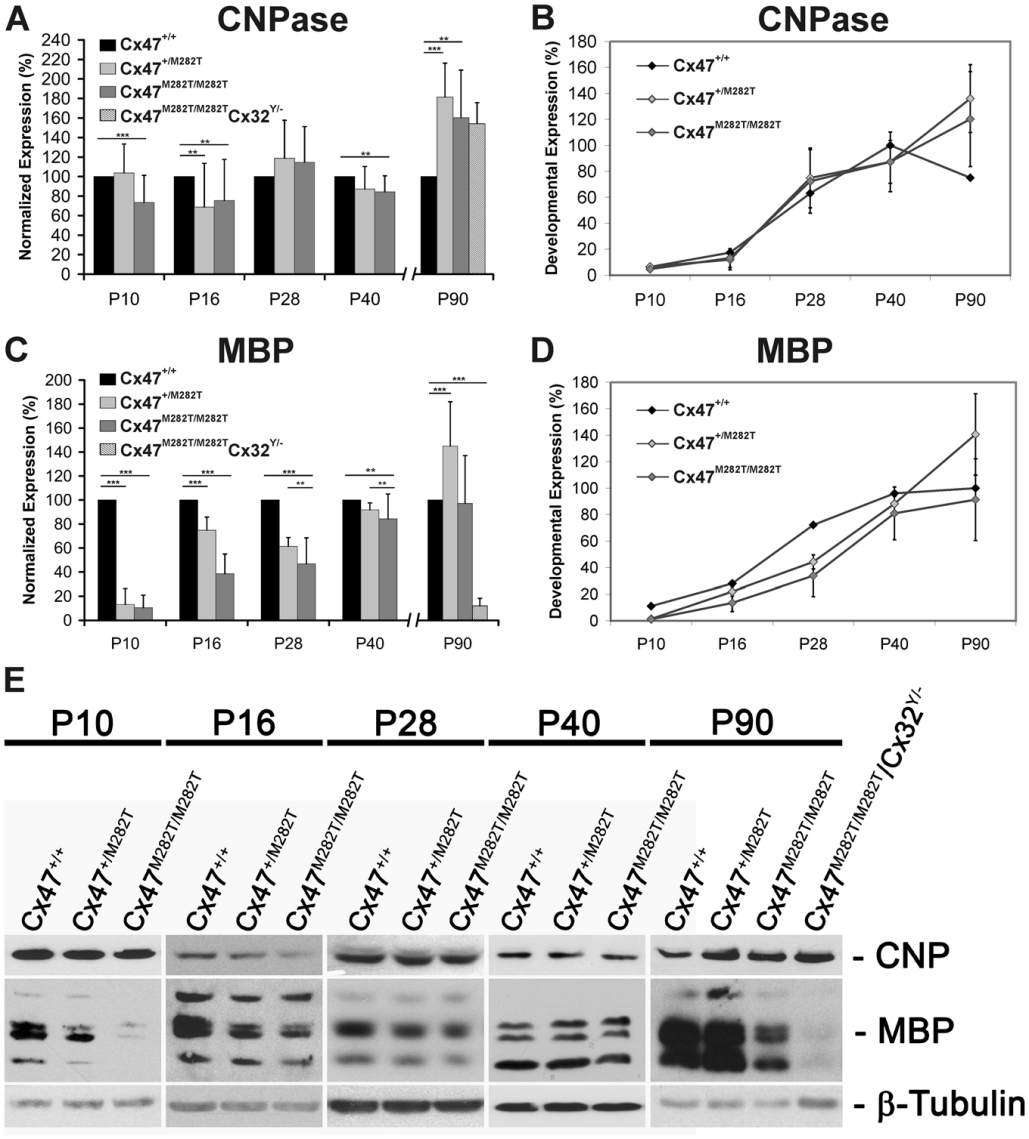
Expression of mutant mCx47*M282T* results in delayed oligodendrocytic differentiation. Immunoblots were performed with cerebellar lysates. At least three animals per group were analyzed. For each oligodendrocytic marker, protein levels were normalized to β-tubulin expression as internal standard. Expression levels of *Cx47^+/+^* mice were set to 100% per group. A, Densitometric quantification of immunoblot analyses revealed significantly decreased CNPase expression in P10, P16 and P40 *Cx47^M282T/M282T^* mice compared to their *wildtype* littermates (** p<0,05; *** p<0,001; ANOVA). *Cx47^+/M282T^* mice showed a significant reduction of CNPase expression on P16 in comparison to *Cx47^+/+^* animals. However, at an age of 90 days both *Cx47^M282T/M282T^* and *Cx47^+/M282T^* mice displayed significantly higher CNPase expression levels compared to *Cx47^+/+^* littermates. Similarly, 90-day-old homozygous mCx47*M282T* expressing mice additionally deficient for Cx32 (*Cx47^M282T/M282T^/Cx32^Y/−^*), displayed significantly increased CNPase levels in immunoblot analysis. B, Relative CNPase expression in *Cx47^+/+^* mice during postnatal development was determined via densitometric analysis of immunoblots. CNPase quantification analyses (A) showed the development-dependent expression of CNPase in cerebella of *Cx47^+/+^*, *Cx47^+/M282T^* and *Cx47^M282T/M282T^* mice. In the cerebellum, *Cx47^+/+^* mice displayed an expression peak of CNPase around P40, while the CNPase levels in *Cx47^+/M282T^* and *Cx47^M282T/M282T^* cerebellar tissue augmented on P90 of postnatal development. C and D, Analogous illustration of MBP expression: C, At each developmental stage, MBP levels in *Cx47^+/M282T^* and *Cx47^M282T/M282T^* cerebella were normalized to the MBP expression level of *Cx47^+/+^* animals (100%). In *Cx47^+/M282T^* and *Cx47^M282T/M282T^* cerebella a significant MBP reduction was observed between P10 and P40 compared to *wildtype* littermates, with strongest impact on P10. Three-month-old *Cx47^M282T/M282T^* mice did not show significantly altered MBP expression any longer, while MBP signals were significantly enhanced in *Cx47^+/M282T^* mice on P90. Immunoblots on lysates obtained from P90 *Cx47^M282T/M282T^/Cx32^Y/−^* mice revealed a clear reduction of MBP expression. D, Development-dependent MBP expression illustrates early hypomyelination and compensation during adolescence in *Cx47^+/M282T^* and *Cx47^M282T/M282T^* animals. Error bars indicate SEM. E, Representative immunoblot analyses on cerebellar tissue obtained from *Cx47^+/+^*, *Cx47^+/M282T^* and *Cx47^M282T/M282T^* mice during postnatal development (40 µg proteins/lane). *Cx47^M282T/M282T^/Cx32^Y/−^* animals analyzed on P90 exhibited disorders during movement.

To ascertain the impact of mCx47*M282T* on oligodendrocyte differentiation, we examined the expression of the early oligodendrocyte marker 2′,3′-cyclic nucleotide 3′ phosphodiesterase (CNPase) and the marker for mature myelinating oligodendrocytes myelin basic protein (MBP) [29] at five stages (P10–P90) of postnatal development (Figure 10).

One way ANOVA of quantitative immunoblot analyses of cerebellar lysates revealed a significant reduction of CNPase in juvenile and young adult *Cx47^M282T/M282T^* mice (P10: 73±28%, n = 5, p<0.001; P16: 76±42%, n = 5, p<0.05 and P40: 84±17%, n = 5, p<0.05), while *Cx47^+/M282T^* mice displayed a significant reduction of CNPase levels to 67±45%, n = 5, p<0.05 only at P16, compared to *wildtype* littermates (Figure 10A, B and E). Analyses on lysates obtaind from P28 mice revealed levels of CNPase for both tansgenic genotypes which were not significantly altered compared to *wildtype* littermates (Cx47^+/M282T^: 119±39%, n = 3, p = 0.26 and *Cx47^M282T/M282T^*: 115±37%, n = 3, p = 0.2). Immunoblot analyses on cerebellar lysates obtained from P90 *Cx47^+/M282T^* and *Cx47^M282T/M282T^* mice revealed significantly elevated expression of CNPase with a mean level of 181±35%, n = 5, p<0.001, for *Cx47^+/M282T^* mice and 160±49%, n = 5, p<0.001, for *Cx47^M282T/M282T^* mice in comparison to *wildtype* animals (100%).

Even more pronounced, expression of MBP was significantly diminished in both *Cx47^+/M282T^* and *Cx47^M282T/M282T^* mice on days P10, P16, P28 and P40 (Figure 10C–E), as compared to *wildtype* littermates. This reduction was particularly obvious in P10 *Cx47^M282T/M282T^* mice (10±10%, n = 5, p<0.001) and in P10 *Cx47^+/M282T^* mice (13±10%, n = 5, p<0.001). Sixteen day-old heterozygous transgenic mice showed less severe but significant MBP reduction to 75±11% (n = 5, p<0.001) and homozygous mCx47*M282T* expressing mice still had intensly reduced MBP levels to 39±17% (n = 5, p<0.001). Similar results were observed for cerebella of 28-day-old mice with a mean reduction of MBP expression to 61±7% (n = 3, p<0.05) in *Cx47^+/M282T^* mice and to 47±22% (n = 3, p<0.001) in cerebella obtained from *Cx47^M282T/M282T^* mice. Although the MBP levels were still significantly reduced, the expression almost reached *wildtype* levels in lysates obtained from 40-day-old *Cx47^+/M282T^* mice (92±6%, n = 5, p<0.05) and *Cx47^M282T/M282T^* mice (84±21%, n = 5, p<0.05). After three months of postnatal development (P90) MBP expression reached *wildtype* levels in cerebella of *Cx47^M282T/M282T^* animals (97±39%, n = 5, p = 0.83), while beeing significantly elevated in *Cx47^+/M282T^* mice at the same developmental stage (145±37%, n = 5, p<0.001).

These data illustrate reduced formation of myelin during adolescence in cerebella of mCx47*M282T* expressing mice, which is compensated during adulthood.

### Deletion of Oligodendrocytic Cx32 in Homozygous *Cx47^M282T/M282T^* Mice Results in Severe Myelin Defects, Astrogliosis, and Microglial Activation

Mice deficient for both Cx47 and Cx32 (*Cx47^−/−^/Cx32^−/−^*) display severe myelin malformations and die on average on day 51 [13]. In order to analyze whether mCx47*M282T* expression is sufficient for proper myelin maintenance or leads to malformations in accordance with *Cx47^−/−^/Cx32^−/−^* mice, we crossbred *Cx47^M282T/M282T^* male and *Cx47^+/M282T^/Cx32^+/−^* female mice to obtain *Cx47^M282T/M282T^/Cx32^Y/−^* male mice. These animals were Cx32-deprived and expressed the mCx47*M282T* mutation homozygously. Like *Cx47^−/−^/Cx32^−/−^* mice, adult *Cx47^M282T/M282T^Cx32^Y/−^* displayed severe CNS myelin defects. Immunoblot analyses of cerebellar lysates obtained from P90 *Cx47^M282T/M282T^Cx32^Y/−^* mice revealed a significant MBP decrease to residual 11.7±7.3% expression compared to *Cx47^+/+^* mice, while CNPase expression was not significantly altered (Figure 10A, 10C and 10E). Furthermore, histochemical analysis yielded only faint MBP signals while increased GFAP and Iba1 expression was obvious in the cerebellar white matter of these mice (Figure 9M, 9N, and 9O). Only three out of eight animals reached three months of age but displayed severe motor impairment. Death of *Cx47^M282T/M282T^Cx32^Y/−^* mice began at the age of 42 days. All dying animals displayed pronounced ataxia one to two days before death. *Cx47^+/M282T^Cx32^Y/−^* littermates did not die and displayed staining patterns similar to *Cx47^+/M282T^* mice (Figure 9J, 9K, and 9L). These data further illustrate that the loss of Cx47 function in vivo is caused by the *M282T* missense mutation.

## Discussion

### Delayed Myelin Formation in Homozygous *Cx47^M282T/M282T^* Mice Is Caused by Loss of Cx47 Function *In Vivo*

Here we have analyzed phenotypic abnormalities of a new transgenic mouse line, carrying a - loss of function - point mutation which leads to human inherited PMLD1. We decided to introduce only the *M282T* missense mutation into the mouse Cx47 gene, in order to avoid side effects of other amino acid differences between mouse and human Cx47 proteins.

Expression of the mCx47*M282T* missense mutation, whose orthologous hCx47*M283T* counterpart was homozygously found in patients, results in a complex but variable neuropathologic phenotype in juvenile, homozygous Cx47*M282T* mice.

Cx47 deficiency as well as homozygous Cx47M282T expression leads to similar phenotypes in mice, suggesting that the point mutation of Cx47 results in loss of function proteins in vivo. Accordingly, biocytin injections into corpus callosum oligodendrocytes participating in glial networks revealed a 88% decrease in number of coupled cells in *Cx47^M282T/M282T^* P10–P15 mice which was very similar to the 85% reduction observed with *Cx47^−/−^* mice. Furthermore, like Cx47 deficiency, homozygous Cx47*M282T* expression caused a significant reduction of the CNPase- and GFAP-negative population of coupled cells within panglial networks. We have previously characterized the majority of this cell population as oligodendrocyte precursors by expression of Olig2, a marker for both immature and mature oligodendrocytes and by activity of the NG2 promoter [23].

Immunofluorescence analyses revealed only few Cx47 positive puncta in brain slices of *Cx47^M282T/M282T^* mice, although the ß-galactosidase reporter protein, which is encoded by the same bicystronic mRNA was robustly expressed. Loss of mutant protein expression due to the bicistronic mRNA construct carrying IRES LacZ, in addition to the Cx47M282T coding sequence was ruled out by immunoprecipitation and subsequent detection via immunoblot. Furthermore, the IRES was introduced 87 nucleotides downstream of the first and 79 nucleotides upstream the second cistron to achieve optimal expression of both coding sequences and similar constructs did not result in loss of expression in recently published mouse lines [30]–[32].

Cell culture experiments revealed that transfectants expressing different Cx47 missense PMLD1 mutations showed accumulation in the endoplasmatic reticulum (ER) of most mutant connexins. This resulted in loss of punctate immunostaining in the plasma membrane and loss of channel function [8], [25]. In addition to ER retention, Cx47*M283T* connexins were occasionally located at the plasma membrane in HeLa cells [25]. We obtained similar results by expression of the orthologous mCx*47M282T* protein in HeLa cells (data not shown). However, we could not identify intracellular accumulations of the mutant protein in vivo and immunosignals in the plasma membrane were very rare in homozygous mice. This could be caused by attenuated signals due to trafficking abnormalities and dispersal of Cx47 connexons or by rapid degradation of the mutant protein, possibly ER associated [33].

Thus, loss of Cx47 function may be due to degradation, rapid internalization and closure of gap junction channels. As a first step to clarify this issue, quantitative Cx47 immunoblots are needed but all currently available antibodies yield prominent unspecific signals in Cx47 deficient brain lysates [34].

### Functional Implications of Cx47 Channels for Myelin Development and Maintenance

Expression of connexins was shown to be critical for cell migration in the CNS [35] but homozygous expression of the mutant mCx47*M282T* did not alter the typical distribution pattern of oligodendrocytes during brain development. On the other hand, ten-day-old homozygous mCx47*M282T* mice displayed a significant reduction of CNPase expression by 28% and more diminished MBP levels (by 90%), whereas 90-day-old mCx47*M282T* expressing mice showed increased levels of CNPase and almost normal levels of MBP. CNP expression appears early during oligodendrocyte differentiation compared to MBP which indicates mature oligodendrocytes in vivo [36], [29]. Thus, our data support the notion that Cx47 function in developing oligodendrocytes is needed for regular oligodendrocyte development and proper timing of myelination rather than for oligodendrocyte migration.

Since outright degenerations of white matter as described in Figure 4 are comparatively rare and appear to follow a highly focal distribution, it is unlikely that they contribute to any major degree to the functional alterations seen in these mice. Rather, they may be seen as reflecting local transport imbalances surpassing a critical lethal threshold, while the functional deficits are caused by more generalized transport deficiencies. This view is in line with data from heterozygous mice, in which these degenerative effects are largely absent despite their functional impairments.

Gap junctional conduits couple apposed cells and may form reflexive channels that connect myelin sheaths in oligodendrocytes and Schwann cells [22], [37]. Panglial networks built by astrocytic and oligodendrocytic gap junctions are considerd to play a key role in spatial K^+^ siphoning after neuronal activity [38]–[40]. Furthermore, gap junctional communication may also be needed to provide oligodendrocytes with metabolites in particular during differentiation and myelin formation. Insufficient supply of metabolites such as glucose, ATP, and possibly N-acetylaspartate (NAA, 173 Da molecular mass) due to loss of Cx47 channel function is likely to play an important role for the hypomyelinating phenotype of Cx47*M282T* expressing mice and may be the cause of PMLD1 in humans. Increased levels of NAA represent a typical feature of Canavan Disease, a fatal dysmyelinating disorder caused by mutations of the oligodendrocytic enzyme aspartoacylase (ASPA) [41]. Most children with Canavan disease die in the first decade of life, while mice with loss of Aspa function are able to survive beyond 12 months but display progressive vacuolation in myelin starting around P14 [42], [43]. PMLD patients showed normal to slightly elevated NAA levels but the N-acetylaspartylglutamate (NAAG) level is increased in the cerebral spinal fluid of PMLD patients harbouring Cx47 mutations [2], [44], [45]. NAAG is released by neurons under depolarizing conditions and degraded by the astrocytic enzyme glutamate carboxypeptidase II (GCP II) to glutamate and NAA [46], [47]. Intraastrocytic NAA may be transported to oligodendrocytes via gap junction channels where it is cleaved by ASPA to aspartate and acetate that may be used subsequently for myelin lipid synthesis [48]. Investigations on lipid composition, NAA, NAAG and aspartate levels in myelin and CSF of *Cx47^−/−^*, *Cx47^M282T/M282T^* and *Cx47^M282T/M282T^/Cx32^−/−^* mice should clarify whether these metabolites play a major role in PMLD1.

Besides loss of channel function, loss of intracellular protein interaction may also be relevant for the phenotype of Cx47M282T expressing mice and for PMLD. Cx47 was shown to interact with the tight junction adaptor protein zonula occludens 1 (ZO-1), the Y-box transcription factor zonula occludens 1 associated nucleic acid binding protein (ZONAB) which regulates expression of the proliferating cell nuclear antigen (PCNA), cyclin D1 and ErbB-2. [49]–[52]. Interaction of ZONAB and ZO-1 is associated with the transition from a proliferative to a differentiated state of epithelial cells [53], [54]. In Cx47 deficient mice punctate staining for ZONAB is lost from oligodendrocyte cell somata while ZO-1 signals remain [50]. Analyses on interactions of these proteins with the mutant Cx47*M282T* using this mouse model will help to gain further insight into intracellular mechanisms which might be influenced by the disease causing mutation.

### Cx32 Expression Largely Compensates for the Loss of Cx47 Protein Function in Adult *Cx47^M282T/M282T^* Mice

Given that myelination in mutant mice was largely coequal to *wildtype* mice six weeks after birth, other oligodendrocytic connexins like Cx29 or Cx32 can probably account for compensatory effects in mice.

In normaly developed myelin Cx29 is not localized at oligodendrocyte somata but mainly at the periaxonal space and was recently shown to be incapable of forming functional intercellular gap junction channels [16], [24], [49], [55]. This indicates a distinct role of Cx29 in myelin function and makes it an improbable candidate for functional redundancy of Cx47 channels.

Mice deficient for both Cx47 and Cx32 (*Cx47^−/−^/Cx32^−/−^*) showed severe myelin abnormalities and premature death [13], [56] suggesting functional redundancy of both connexins. This led to the speculation that mutations of Cx47 might lead to a detrimental gain of function and inhibition of Cx32 gap junctional channels [1].

Although homozygous Cx47*M282T* expression results in diminished or total loss of Cx47 gap junction channel function, it does, like Cx47 deficiency, not lead to a complete inhibition of interoligodendrocytic coupling as recently described for *Cx47^−/−^/Cx32^−/−^* mice [23]. Furthermore, death of *Cx47^M282T/M282T^/Cx32^−/−^* animals began 42 days after birth and 90-day-old *Cx47^M282T/M282T^/Cx32^−/−^* mice displayed severe motor impairment, myelin malformations, vacuolation, pronounced astrogliosis and increased Iba1 expression, whereas P90 *Cx47^M282T/M282T^* mice showed minor affected myelin in the cerebellum. These data further corroborate that the *M282T* missense mutation results in loss of Cx47 channel function and underline the common role of Cx32 and Cx47 channels for myelin maintenance. In addition, these data show that Cx47*M282T* does not have a transdominant negative effect on Cx32 function *in vivo*.

Detailed immunofluorescence analyses have revealed a drafmatic increase of Cx32 levels along myelinated fibers in the CNS of Cx47^−/−^ mice [50]. It seems probable that increased Cx32 expression or altered Cx32 channel localization can account for loss of Cx47 function compensation in *Cx47^M282T/M282T^* and Cx47 null mice but not in human PMLD patients.

### Heterozygous *Cx47^+/M282T^* Mice Show Unexpected Phenotypic Abnormalities

Although PMLD1 is a recessive disease, heterozygous mCx47*M282T* expressing mice revealed an unexpected CNS phenotype. MBP expression was decreased, accompanied by mild astrogliosis in cerebella of ten-day-old mice. Furthermore, the number of oligodendrocytes forming networks was significantly reduced in heterozygous mice which was not detected in *Cx47^M282T/M282T^* or *Cx47 null* mice. Compared to *wildtype* littermates, adult *Cx47^+/M282T^* but not *Cx47^M282T/M282T^* mice showed significantly decreased locomotion, running speed and reduced rearing behavior in the open field test, but no significant alterations in the rotarod assay suggesting impaired neuronal function in motor circuits, independent of a cerebellar phenotype. Immunofluorescence analyses revealed a reduction of Cx47 positive puncta in myelin of *Cx47^+/M282T^* mice and the number of oligodendrocytes forming intercellular networks was also reduced in these mice. This reduction may have caused the mild myelin abnormalities and alterations in intercellular coupling found in young heterozygous mice. In contrast to *Cx47^M282T/M28T^* mice, high-resolution light microscopy based upon 1 µm thin sections did not reveal lesions in the cerebellum and at best very minor alterations in the optic fascicle. These findings indicate that the functional alterations seen in heterozygous mice are unrelated to myelin degeneration.

Furthermore, *Cx47^+/M282T^/Cx32^−/−^* mice did not die during adolescence and did not show the strong phenotype observed with *Cx47^M282T/M282T^/Cx32^−/−^* and *Cx47^−/−^/Cx32^−/−^* mice. This indicates that residual expression of wildtype Cx47 protein is sufficient to prevent early death in *Cx47^+/M282T^/Cx32^−/−^* mice, in accordance with recessiveness of human PMLD1.

The cause of the phenotypic abnormalities found in heterozygous mice is still enigmatic, but they suggest a gain of detrimental function by heterozygous *Cx47M282T* expression, possibly due to inhibited or disturbed adapter protein interaction or hemichannel dysfunction [8]. Furthermore, the phenotypes observed with heterozygous and homozygous animals were not completely congruous and some abnormalities aggravated with heterozygous expression. This suggests that compensatory effects in homozygous mice are lacking in heterozygous mice, at least in distinct cells.

### Young Homozygous *Cx47^M282T/M282T^* Mice As a Model Organism for PMLD1

The course of PMLD1 is progressive in humans in contrast to myelin pathology in *Cx47^M282T/M282T^* and *Cx47^−/−^* mice. One major difference between mouse and human CNS is the higher portion of white matter in the human brain. While in humans more than 50% of the volume consists of white matter, in the mouse it is only around 10%. The increased number of oligodendrocytes and their relatively extended distance to blood vessels may be one reason for the higher sensibility of the human brain regarding loss of Cx47 function. Furthermore, previous studies have demonstrated the strong ability of the mouse CNS to efficiently compensate for loss of myelin proteins e.g. PLP1 and Nogo-A, MAG or complete elimination of oligodendrocytes during the first weeks of postnatal life [57]–[59]. *Cx47^M282T/M282T^* and *Cx47^−/−^* mice apparently compensate for loss of Cx47 function within the critical time interval before axonal loss occurs [60].

Altogether, our results support the notion that loss of Cx47 channel function causes PMLD1. Furthermore, we consider young homozygous *Cx47M282T* mice, although displaying only transient myelin malformation, as a suitable mouse model to gain closer insights in the mechanisms that lead to inherited human PMLD1.

## Materials and Methods

All mice used in this study were kept under standard housing conditions with a 12 h/12 h dark–light cycle and with food and water ad libitum. All experiments were carried out in accordance with local and state regulations for research with animals. All animals investigated harboured at least 96% C57BL/6 genetic background.

### Cloning of the m*Cx47M282T* Targeting Vector

The hCx47*M283T* mutation from a human patient [1] was inserted by PCR mutagenesis in the orthologous mouse gene resulting in the mCx47*M282T* mutation, cloned into the pBluescript vector and sequenced in both directions by LGC Genomics (Berlin, Germany). The mutated gene was cloned into a vector containing the IRES and the nls-*LacZ* cassettes by *Bcl*I/*Hin*dIII and *Nsi*I digestions and subsequent ligation of blunted ends. The IRES sequence (Clontech Laboratories, CA, USA) was cloned by *Sma*I digestion from IRES_pBluescript [30], into pBS_nls_*LacZ*pA [61] digested with *Hin*dIII and subsequent blunt end generation by Klenow polymerase fill in.

The 17.6 kb targeting vector included two homologous regions with a 4667 bp 5′ intron fragment upstream and a 2479 bp 3′ fragment downstream the Cx47 coding DNA. The neomycin selection cassette including the phospoglycerate kinase promoter was flanked by frt sites and cloned into the 3′ homologous region downstream of the Cx47 polyA signal by *Sal*I/*Xho*I ligation.

The final vector was partially sequenced by LGC Genomics (Berlin, Germany) after restriction analysis. The functionality of the frt sites was verified by transformation into Flp recombinase expressing *E. coli* bacteria [62]. The functionality of the mCx47*M282T*-IRES-nls_*LacZ* construct was tested in HeLa cells (not shown).

### Screening of ES Cell Clones for Homologous Recombination

ES cell culture and transfection were performed as described previously [63]. The targeting vector DNA (200 mg) was linearized by *Sal*I digestion prior to transfection. Neomycin resistant ES cell clones were screened for homologous recombination by PCR using an external primer specific to the Cx47 3′ region (Cx47-3-rev: CCA GGA TTC ATG TGA AGG AGA AGG G) and an internal primer specific to the neomycin resistance cassette (primer C1: CTC TGA GCC CAG AAA GCG AAG GAG). To exclude clones in which recombination had occurred partially, a second PCR analysis was performed with cells positive in the first one. Here, we used primers annealing in the coding region of Cx47 (primer B1: GAG GAG CGA GCG GAG GAT GTG GCT G and primer B2: GTG GCG CTG CCG GTT CCG GAA GCT AG) which flanked the *Bsm*BI restriction site that was introduced by the mutation of the Cx47 gene. *Bsm*BI digestion of the 700 bp PCR fragments yieded additional 350 bp fragments which indicated integration of DNA coding for the mutant mCx47*M282T*. Homologous recombined ES cell clones were subsequently confirmed by Southern blot hybridization. Therefore, DNA from the PCR-positive clones was digested with *Bam*HI or *Eco*RV and DNA fragments were separated via agarose gel electrophoresis. After blotting onto Hybond N+ membranes (Amersham Biosciences, Buck, UK) and ultraviolet crosslinking for fixation, hybridization was performed under stringent conditions using the Quick Hyb solution (Stratagene, La Jolla, CA, USA) at 68°C for 1.5 h. A 454 bp *Bgl*II fragment was used as a 5′ external probe and hybridized with *Bam*HI digested DNA fragments. *Eco*RV digested DNA was hybridized with an 824 bp 3′ external probe generated by *Bam*HI/*Xmn*I digestion and with a 624 bp *Hpa*I Fragment obtained from the *LacZ* coding region serving as an internal probe.

### Generation and Genotyping of the mCx47*M282T* Mice

The homologously recombined ES cell clones were injected into C57BL/6 blastocysts, as described previously [63]. Blastocyst injections of ES cells resulted in fur-color chimeric mice. Breeding of chimeric mice with C57BL/6 mice yielded agouti offspring and germline transmission of the recombinant allele was checked by PCR analyses of isolated tail DNA. Heterozygous *Cx47^+/M282T^neo* mice were crossed to *hACTB:FLPe* mice expressing the Flp-recombinase and litters were backcrossed to C57Bl/6 mice to generate *Cx47^+/M282T^* mice. The heterozygous *Cx47^+/M282T^* mice were backcrossed with C57BL/6 mice to increase the C57BL/6 genetic background. Genotyping of mice was performed by three primer PCR (primer A1: GCA GCA GAG ACG GCA AGG CCA CC, primer AC2: CCG GTC GCT ACC ATT ACC AGT TGG and primer A3: CAG AGA GAG GAG CTG TTC TTG GTC C) and deletion of the frt site flanked neomycin resistance cassette was verified by PCR using primer C2: CTC AGC TGC AGT AAG GGA TCT CTC G, primer C1 and primer AC2 (described above). Presence of the mutation was verified by PCR and subsequent *Bsm*BI digestion as described for screening of ES cell clones. The different allelic variants were further verified by Southern blot analyses on *Mfe*I digested DNA and hybridization with an 845 bp *Eco*RI/*Xho*I fragment as a 3′ internal probe.

### β-Galactosidase Staining and Combined Immunolabelling

Mice were killed by injecting an overdose of anesthetic (combination of ketamin hydrochloride and xylazine hydrochloride) intraperitoneally and subsequent transcardially perfusion with 40 ml phosphate-buffered saline (PBS) followed by 25 ml 2% phosphate-buffered formaldehyde solution (Roti-Histofix; Roth, Karlsruhe, Germany) containing 0.2% glutaraldehyde. Tissues of interest were dissected from the mice and postfixed for 24 h at 4°C. 50 µm sagittal vibratome (VT 1200 S, Leica, Wetzlar, Germany) sections were obtained from brains of Cx47^M282T/M282T^ mice for investigation of development-dependent *LacZ* expression. For immunohistochemistry combined with β-gal staining samples were cryoprotected with 30% sucrose in PBS over night and 40 µm sections were cut with a cryostat (Microm, Walldorf, Germany) and collected on SuperFrost Ultra Plus slides (Menzel, Braunschweig, Germany). β-Gal staining was performed using the substrate 5-bromo-4-chloro-3-indolyl-b-galactoside (X-Gal) as previously described [64]. For doublelabeling immunohistochemical analyses, sections were stained overnight with X-Gal, exposed to PBS containing 0.1% H_2_O_2_ and 10% methanol for 20 min, blocked in M.O.M. reagent (Vector Laboratories, Burlingame, CA, USA) and incubated over night at 4°C with primary antibodies in PBS containing 4% BSA. As primary antibodies monoclonal mouse anti-neuronal nuclei (NeuN; 1∶100, Chemicon, Millipore GmbH, Schwalbach/Ts., Germany), monoclonal mouse anti-CNPase (1∶200, Sternberger Monoclonals) and monoclonal mouse anti-GFAP (1∶400, Sigma-Aldrich) were used. Immunohistochemical analyses were performed using the M.O.M. kit, according to manufacturer's instructions. Development of the peroxidase reaction was carried out using 3,3′-diaminobenzidine tetrahydrochloride (Sigma-Aldrich) as a substrate. Slices were air dried and mounted with Entellan (Merck Chemicals, Darmstadt, Germany).

### Immunofluorescence Labelling

For double immunofluorescence labelling mice were transcardially perfused with PBS containing 1% formaldehyde and 0.1% picric acid, and brains were postfixed in 1% phosphate buffered formaldeyde solution for 2 h and cryoprotected with 30% sucrose in PBS over night. 16 µm cryosections were blocked with TBS (50 mM Tris, 150 mM NaCl, pH 7.5) containing 0.3% Triton X-100 and 5% NGS for 1 h at room temperature. Sections were incubated with both primary antibodies: mouse monoclonal anti-Cx47 (Zymed, 1∶250) and polyclonal rabbit anti-β-gal (ICN, 1∶500) at 4°C over night, washed with TBST (50 mM Tris, 150 mM NaCl, pH 7.5, 0.1% Triton X-100) and incubated for 1 h with appropriate goat polyclonal secondary antibodies conjugated to Alexa-488 and Alexa-594 (1∶500, Invitrogen, Karlsruhe, Germany) diluted in TBS containing 0.3% Triton X-100 and 5% NGS. After TBS washes, sections were mounted with PermaFluor (Thermo Fisher scientific, Waltham, MA, USA) and Images were taken with a Laser Scanning Microscope (LSM 510, Zeiss, Germany).

### Dye Coupling in Corpus Callosum Slices

Dye coupling experiments were performed in parallel with experiments previously described [23] and we shared the controls between these two studies. Acute coronal brain slices containing the corpus callosum were prepared from postnatal days (P) 10–15 mice as previously described [65]. For patch clamp recordings slices were placed in a recording chamber and perfused continuously with artificial cerebrospinal fluid (aCSF) composed of (in mM): NaCl 134; KCl 2.5; MgCl_2_ 1.3; CaCl_2_ 2; K_2_HPO_4_ 1.25; NaHCO_3_ 26; D-glucose 10 and saturated with carbogen (95% O_2_, 5% CO_2_) to a pH of 7.4 at room temperature. Patch clamp recordings were performed as previously described [23]. Pipettes had a resistance ranging from 3 to 7 MΩ when filled with an intracellular solution containing (in mM): NaCl 4; KCl 120; MgCl_2_ 4; CaCl_2_ 0.5; Hepes 10; EGTA 5; D-glucose 5; 0.5% biocytin at pH 7.4. Alexa Fluor 594 (10 µg/ml, Invitrogen, Karlsruhe, Germany) was added to the pipette solution to confirm intracellular access. To improve voltage-clamp control, capacitance was compensated by TIDA software (HEKA Elektronik, Lambrecht, Germany). In each individual slice only a single cell was filled via the patch pipette during whole-cell recordings (20 min) [66]. Only cells with stable input resistance over the 20 min period were considered for data analysis. During recording, the membrane was continuously de- and hyperpolarized between −170 and + 50 mV from a holding potential of −70 mV (10 mV steps, 50 ms). Current signals were amplified (EPC 9/2 or EPC10 amplifiers, HEKA), filtered (3 kHz), sampled (5 kHz), and monitored with TIDA software (HEKA).

### Immunohistochemistry following Dye Coupling Experiments

After recording, slices were fixed and processed for biocytin visualization with fluorochrome conjugated streptavidin combined with immunostaining for the oligodendrocyte marker CNPase and the astrocytic marker GFAP, as previously described [23]. Cy3-conjugated streptavidin (1∶200, Jackson ImmunoResearch/Dianova, Hamburg, Germany), mouse anti-CNPase (1∶200, Covance/HISS Diagnostic GmbH, Freiburg, Germany) and rabbit polyclonal anti-GFAP (1∶1.000, DAKO, Hamburg, Germany) were used. Primary antibodies were visualized by application of FITC-conjugated donkey anti-mouse IgG (1∶200) and Cy5-conjugated donkey anti-rabbit IgG (1∶200) or by FITC-conjugated donkey anti-chicken IgG (1∶200, secondary antibodies were purchased at Jackson ImmunoResearch/Dianova), respectively. No unspecific cross reaction between secondary antibodies was observed. Images were acquired by confocal microscopy (Leica TCS SP5, Leica, Solms, Germany) with Leica software (LAS AF Lite).

### Evaluation of Dye Coupling

Electrophysiological data were analyzed and plotted using TIDA and Origin (MicroCal, Northampton, Massachusetts). Biocytin filled cells were counted on sequential confocal stacks (0.2–0.8 µm steps) of the 110 µm thick slices with Image-Pro plus (MediaCybernetics, L.P., Bethseda, MD, USA). The maximum extent of tracer spread, defined as the largest distance between two somata within a network, was measured with the same software used for image acquisition. Statistic analysis was performed with SPSS 11.5 for Windows (SPSS Inc., Chicago, Illinois). The cohort of controls used was the same as previously published since the dye coupling experiments described here were performed in parallel with the former study [23]. Differences between groups were evaluated with the Kruskal-Wallis nonparametric test followed by Mann-Whitney U-test for two independent samples, Bonferroni corrected for n-pair comparisons. All values are expressed as median, 25^th^ and 75^th^ percentiles. Values of specific glial cell types are given as percentage of the number of biocytin-positve cells per network. For comparisons between groups, the number of oligodendrocytes forming networks was evaluated with Chi Square-test. Data regarding the extent of tracer spread are expressed as mean ± standard deviation (SD) and were analyzed with one-way ANOVA, followed by Bonferroni *post hoc* analysis. P-values of <0.05 were considered statistically significant.

### Immunoblot and Cx47 Immunoprecipitation

Mice were killed by cervical dislocation and whole brain or cerebella were quickly dissected out and flash frozen in liquid nitrogen. Tissues were stored at −80°C until extraction in homogenization buffer (20 mM Tris, 1% Triton X-100, 140 mM NaCl, 10% glycerol, 1 mM EGTA, 1.5 mM MgCl_2_, pH 8.0) containing protease inhibitors (Complete Mini; Roche Diagnostics). The samples were homogenized on ice with a glas pestle and subsequently sonicated two times for 15 s. After centrifugation for 15 min at 10.000 g and 4°C, the supernatant was retrieved in new 1,5 ml tubes and kept at −80°C or −20°C. HeLa-Cx47-eGFP [67] cell lysates were prepared in homogenization buffer. Cultured HeLa cells were harvested and lysed as described previously [68]. Samples were denatured at 60°C for 10 min (Cx47) or 95°C for 5 min containing 1× Laemmli buffer. Proteins (40–100 µg) were separated by electrophoresis on a 10–15% polyacrylamide gel and transferred to Hybond ECL membrane (Amersham Bioscience). Blots were preincubated in a blocking solution of 5% milk powder (MP) in TBST (50 mM Tris, 150 mM NaCl, pH 7.5, 0.1% Tween-20) for 1 h at room temperature, incubated with primary antibodies overnight at room temperature (5 h with anti-Cx47 antibodies) and after three wash steps, with horseradish peroxidase (HRP)-conjugated antibodies (1∶2.000–1∶10.000, Dianova, Hamburg, Germany).

Primary antibodies were polyclonal rat anti-MBP (1∶1.000 in 5% MP, Chemicon, Millipore GmbH, Schwalbach/Ts., Germany), monoclonal mouse anti-CNPase (1∶2.000 in Roti-block (Roth, Karlsruhe, Germany), Sigma-Aldrich) rabbit anti-Cx47 (1∶250 in 4% BSA, No. 36-4700, Invitrogen, Karlsruhe, Germany), rabbit anti β-gal (1∶500 in 5% MP, ICN) and monoclonal mouse anti-tubulin (1∶10.000 in 5% MP, Chemicon, Millipore GmbH, Schwalbach/Ts, Germany).

Protein bands were detected by incubation of the membranes with enhanced chemiluminescence reagents (Amersham Bioscience) and development on X-ray films. Densitometry analysis was performed with E.A.S.Y Win32 software and by normalizing the band intensities to tubulin values.

For immunoprecipitation, 250 µg protein of HeLa-Cx47-eGFP lysate and 600 µg protein obtained from whole brain lysate of *wildtype Cx47^+/+^*, *Cx47^−/−^* and *Cx47^M282T/M282T^* mice were incubated with 85 µl protein A sepharose CL-4B (Amersham Bioscience) each for 3 h at 4°C in TBS and subsequently centrifuged (12.000 g, 4°C, 30 min). Mouse Cx47 antibodies (No. 37-4500, Invitrogen, Karlsruhe, Germany, 2.5 µg) were incubated with 25 µl of protein A-Sepharose (Amersham Biosciences) on ice for 2 h. The precleared lysates were incubated with the protein A-Sepharose antibody complex overnight at 4°C and washed three times with TBST. The proteins were eluted in 18 µl of Laemmli buffer (60°C, 10 min) and separated by electrophoresis as stated above.

### Histology

Mice were killed with an overdose of chloroform and sequentially perfused via the left ventricle with phosphate buffer with 1% procaine HCL and then 6% glutardialdehyde in phosphate buffer. Tissues of interest were dissected from the mice and embedded in epoxy resin (Epon 812/glycid ether) after postfixation in 2% OsO_4_. Sections were cut at a thickness of 1 µm heatmounted on aminosilane-coated slides and stained with toluidine blue and pyronin G.

### Immunohistochemical Staining on Vibratome Sections

At different ages mice were killed by injecting an overdose of anesthetic intraperitoneally. Mice were transcardially perfused with 40 ml phosphate-buffered saline (PBS) followed by 25 ml phosphate-buffered formaldehyde solution 4% (Roti-Histofix; Roth, Karlsruhe, Germany). The brains were rapidly prepared and postfixed in 2% phosphate-buffered formaldehyde solution for at least 48 h at 4°C. 25 µm vibratome sections were obtained (VT 1200 S, Leica, Wetzlar, Germany) and free floating slices were incubated with 0.1% H_2_O_2_ in 10% methanol/PBS (pH 7.4) for 30 min to inhibit endogenous peroxidase activity, washed in PBS and incubated in blocking solution (5% normal goat serum (NGS), 4% BSA, 0.3% Triton X-100, 0.01% NaN_3_ in PBS) for 2 h at room temperature to avoid unspecific crossreactivity. Primary antibodies were applied in blocking solution for 16 h at room temperature. After washing with 0.2% Triton X-100 in PBS, sections were incubated with the appropriate biotin-conjugated secondary antibody for 2 h at room temperature and washed again. Vectastain Peroxidase ABC reagent (Vector Laboratories, Burlingame, CA, USA) was applied following the manufacturer's protocol. After 30 min incubation in working solution free floating sections were washed in 0.05 M Tris for 20 min and then transfered to bidest for at least 5 min prior to NovaRed (Vector Laboratories, Burlingame, CA, USA) staining. Sections were mounted on glass slides, air dried at 45°C on a slide warmer and coverslipped with Entellan (Merck Chemicals, Darmstadt, Germany). As primary antibodies rat anti-MBP antibodies (1∶750, Chemicon, Millipore GmbH, Schwalbach/Ts, Germany) were used for myelin staining, rabbit polyclonal anti-Iba1 antibodies (1∶750, Wako Chemicals GmbH, Neuss, Germany) were used for staining of microglia and rabbit polyclonal anti-GFAP antibodies (1∶1.000, Dako, Carpinteria, CA USA) were used for detection of astrocytes.

### Behavioral Analyses

The mice were housed individually in macrolone cages (Type 2, 22×16×13 cm) with metal covers and were given free access to standard rodent diet (Ssniff, Spezialdiäten GmbH, Soest, Germany) and water. They were maintained on a 12 h light-dark schedule with lights on at 7 a.m. and were tested during the light phase between 9 a.m and 6 p.m. All experiments were approved by the North Rhine Westphalia State Authority in accordance with the German legislation on animal experimentation (German Animal Welfare Act, TSchG).

### Open-Field Test

Each animal was subjected to three trials in the open-field with an inter-trial interval of 24 h. The open-field was constructed of grey plexiglas and had the following dimensions: 30 cm (height)×30 cm (length)×30 cm (width). It was illuminated by diffuse white light providing a low illumination density of approximately 10 lux at the center and was placed in a sound-attenuated chamber. A camera mounted above the open field transmitted the digitized image of the open-field to a computer and a VCR. The base area of the open field was subdivided by software into nine equal sized quadrants, providing a 10×10 cm central quadrant and four corner quadrants. Each mouse was individually placed into the central quadrant of the open-field and allowed to freely explore the arena for 10 min. After each trial, the entire apparatus was cleaned with 50% ethanol solution and dried thoroughly. A semi-automated video tracking system (Ethovision Software, Noldus, The Netherlands) was used to quantify the animal's horizontal (the distance travelled in cm, mean running speed in cm/s), and vertical activity (number and duration (s) of rearings on the hind limbs).

### Motor Coordination and Balancing on the Rotarod

Motor coordination and balancing was tested with an accelerating rotarod (TSE systems; Bad Homburg, Germany; model no.: 7650). The rotating rod was elevated 10 cm off the floor, had an axis diameter of 3.5 cm and a striated surface made of black rubber. During the acquisition phase, each mouse was given three trials (with an inter-trial interval of ≥25 to control for possible effects of physical exhaustion) per day for three consecutive days. On each trial, the mouse was placed on the inactive drum, with its head pointing towards the direction opposite to that of the rod's motion. The mouse had to move forward on the drum, which was rotating along its vertical axis, in order to avoid falling off. Over a period of 300 s, the rod step-wise accelerated to a speed of 50 rpm. As some mice tend to passively ride around the rod, especially at higher velocities, the duration (s) of active performance until the mouse fell off the drum was registered with a cutoff after 300 s. After retention delay of one week the long-term motor memory of the animals was evaluated during three trials.

### Statistical Analysis

Data are presented as mean ± standard error of mean (SEM). Data were analyzed by repeated measures ANOVA and by pre-planned Bonferroni tests between transgenic and wild-type mice. P-values lower than 0.05 were considered statistically significant.

## Supporting Information

Figure S1 A variable number of blood vessels predominantly in the hippocampal region of *Cx47^M282T/M282T^* mice feature perivascular glia being filled with basophilic material or undergoing conspicuous destruction (*Cx47^+/+^* shown in A, C; *Cx47^M282T/M282T^* in D and E, arrows pointing to glia cells). Additionally, scattered neurons within the pyramidal cell layer show similar degenerative changes (*Cx47^+/+^* in A, *Cx47^M282T/M282T^* in B). Scale bars: A–C, 20 µm; D and E, 10 µm.
(TIF)
