# Supplementary figures and images for: Pathologic and Phenotypic Alterations in a Mouse Expressing a Connexin47 Missense Mutation That Causes Pelizaeus-Merzbacher–Like Disease in Humans
Source: PLoS Genet. 2011 Jul 7;7(7):e1002146. doi: 10.1371/journal.pgen.1002146 (PMC3131295; doi:10.1371/journal.pgen.1002146)

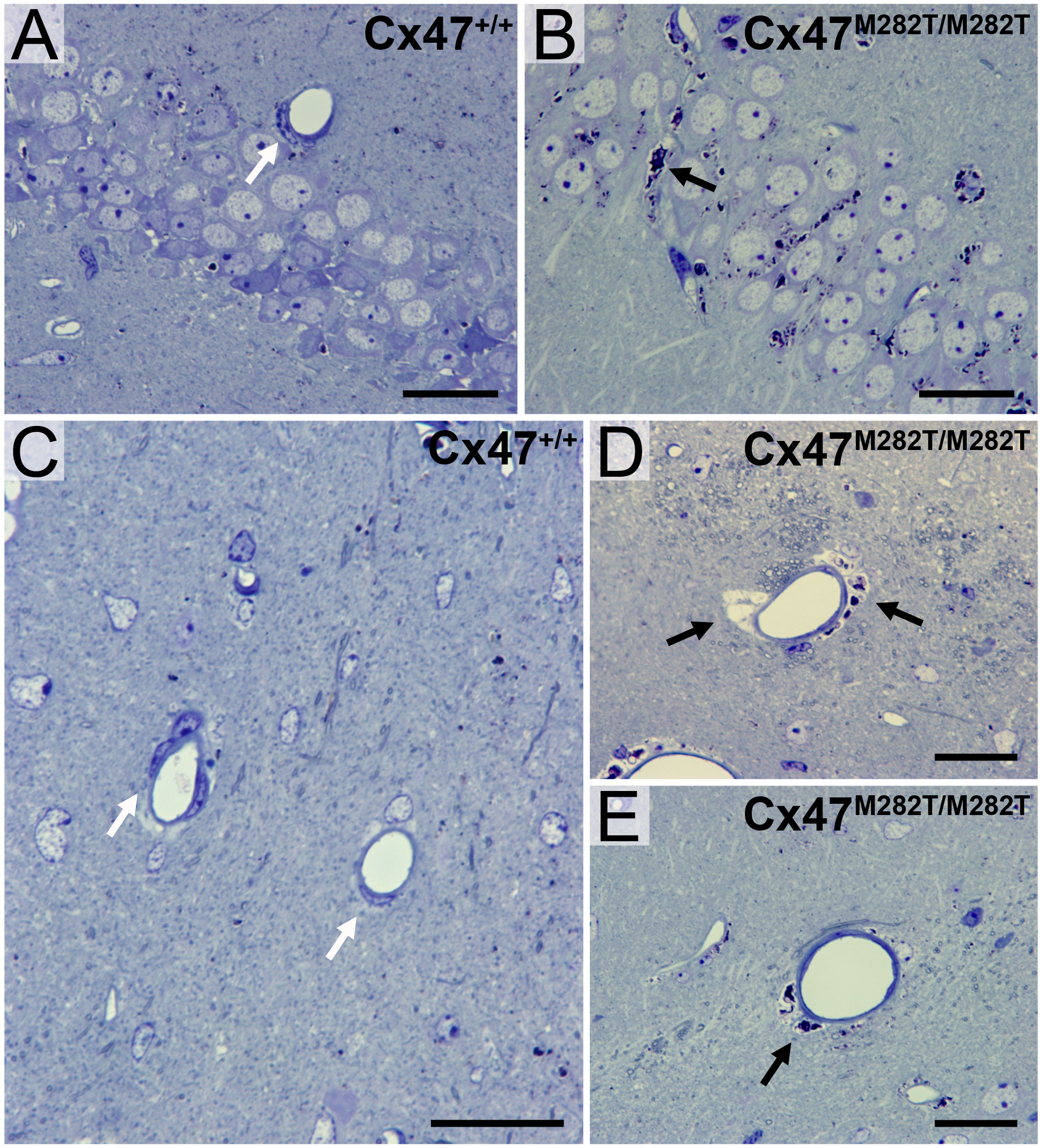

Supplement: Figure S1 — A variable number of blood vessels predominantly in the hippocampal region of Cx47M282T/M282T mice feature perivascular glia being filled with basophilic material or undergoing conspicuous destruction (Cx47+/+ shown in A, C; Cx47M282T/M282T in D and E, arrows pointing to glia cells). Additionally, scattered neurons within the pyramidal cell layer show similar degenerative changes (Cx47+/+ in A, Cx47M282T/M282T in B). Scale bars: A–C, 20 µm; D and E, 10 µm. (TIF) [file pgen.1002146.s001.tif]
